# Tumor-specific activation of folate receptor beta enables reprogramming of immune cells in the tumor microenvironment

**DOI:** 10.3389/fimmu.2024.1354735

**Published:** 2024-02-07

**Authors:** Fenghua Zhang, Bo Huang, Sagar M. Utturkar, Weichuan Luo, Gregory Cresswell, Seth A. Herr, Suilan Zheng, John V. Napoleon, Rina Jiang, Boning Zhang, Muyi Liu, Nadia Lanman, Madduri Srinivasarao, Timothy L. Ratliff, Philip S. Low

**Affiliations:** ^1^ Department of Chemistry and Institute for Drug Discovery, Purdue University, West Lafayette, IN, United States; ^2^ Purdue University Institute for Cancer Research, Purdue University, West Lafayette, IN, United States; ^3^ Department of Comparative Pathobiology, Purdue University, West Lafayette, IN, United States; ^4^ Department of Medical Oncology, Dana-Farber Cancer Institute, Boston, MA, United States; ^5^ University of North Texas Health Science Center at Fort Worth, Fort Worth, TX, United States; ^6^ Department of Computer Sciences, Purdue University, West Lafayette, IN, United States

**Keywords:** folate receptor beta, tumor associated macrophages, myeloid derived suppressor cells, reprogramming of tumor microenvironment, single-cell RNA-seq analysis

## Abstract

Folate receptors can perform folate transport, cell adhesion, and/or transcription factor functions. The beta isoform of the folate receptor (FRβ) has attracted considerable attention as a biomarker for immunosuppressive macrophages and myeloid-derived suppressor cells, however, its role in immunosuppression remains uncharacterized. We demonstrate here that FRβ cannot bind folate on healthy tissue macrophages, but does bind folate after macrophage incubation in anti-inflammatory cytokines or cancer cell-conditioned media. We further show that FRβ becomes functionally active following macrophage infiltration into solid tumors, and we exploit this tumor-induced activation to target a toll-like receptor 7 agonist specifically to immunosuppressive myeloid cells in solid tumors without altering myeloid cells in healthy tissues. We then use single-cell RNA-seq to characterize the changes in gene expression induced by the targeted repolarization of tumor-associated macrophages and finally show that their repolarization not only changes their own phenotype, but also induces a proinflammatory shift in all other immune cells of the same tumor mass, leading to potent suppression of tumor growth. Because this selective reprogramming of tumor myeloid cells is accompanied by no systemic toxicity, we propose that it should constitute a safe method to reprogram the tumor microenvironment.

## Introduction

Infiltrating immune cells and fibroblasts are major components of solid tumors that can become increasingly immunosuppressive as their residence times in tumor masses prolong (1–8). Although peripheral blood myeloid cells are predominantly nonpolarized prior to tumor entry (8), they can release immunosuppressive cytokines (7, 9) (e.g. TGFβ, IL-10), secrete cancer proliferating growth factors (7, 10, 11) (e.g. EGF, HGF, PDGF, VEGF), inactivate cytotoxic T cells via T cell receptor nitrosylation (1, 8), exude nucleotide bases that facilitate cancer cell mitosis (12), promote tumor metastasis (6, 13, 14), and stimulate extracellular matrix deposition that can obstruct drug and immune cell penetration (15) following accumulation in tumor masses. Although many strategies have been explored to suppress these tumor-supporting activities (9, 16), they have generally been impeded by unwanted toxicities associated with stimulation of the immune system in healthy tissues.

Folate receptor beta (FRβ) has recently attracted considerable attention as a biomarker of tumor infiltrating macrophages, not only because its expression is restricted to myeloid cells (5), but also because FRβ is generally found to be upregulated on immunosuppressive myeloid derived suppressor cells (MDSCs) and tumor associated macrophages (TAMs) (6–8). We have recently shown that a subset of these FRβ+ TAMs and MDSCs can be repolarized from a tumor-promoting to tumor-suppressing phenotype with FRβ-targeted drugs (8, 17). Because this reprogramming was achieved without perturbing the properties of myeloid cells in healthy tissues, the question arose whether FRβ might be somehow different in myeloid cells from healthy and malignant tissues. We demonstrate here that FRβ expression is restricted to a small subset of myeloid cells in healthy tissues and that in these healthy tissues FRβ+ is functionally inactive. We further show that incubation of these same healthy myeloid cells in either cancer cell conditioned media or anti-inflammatory cytokines (IL-4 plus IL-13) induces a change in FRβ functionality from a nonbinding to folate-binding state, and that this activation of FRβ enables the receptor to bind and internalize folate-targeted drugs with high (~1 nM) affinity. We finally employ single-cell RNA-seq to characterize the changes in gene expression associated with the selective repolarization of TAMs and MDSCs by a folate-targeted drug and demonstrate that the tumor infiltrating myeloid cells not only change from an M2- to M1 like phenotype, but that they in turn induce a proinflammatory shift in the phenotypes of most other immune cells in the tumor microenvironment.

## Methods

### Cell culture

THP-1 cells (Cat# TIB-202), 22RV1 cells (Cat# CRL-2505), MDA-MB-231 cells (Cat# CRM-HTB-26), Raw264.7 cells (Cat# TIB-71) and 4T1 cells (Cat# CRL-2539) were purchased from ATCC. Unless otherwise specified, cells were cultured in folate-free RPMI 1640 medium (Gibco, Ireland) containing 10% heat-inactivated fetal calf serum and 1% penicillin-streptomycin in 5% CO_2_ at 37°C. When cells reached confluence, spent medium was harvested and centrifuged to remove cells and debris. The resulting supernatant was filtered twice through a sterile 0.2μm cell strainer and then used to differentiate M0 into M2 macrophages as described below.

### Induction of cytokine biosynthesis in healthy human blood and Raw264.7 cells by TLR7-1A and FA-TLR7-1A

Fresh whole human blood was collected from healthy donors and was either used directly for cytokine stimulation studies or isolation of peripheral blood mononuclear cells (PBMCs) using density gradient media (Ficoll-Paque PLUS, Cat# 17144002) as described previously (18). The isolated monocytes were differentiated into M0-like macrophages by incubation with 20ng/ml human M-CSF for 7 days (18), after which the M0-like macrophages were polarized into M2-like macrophages by further incubation for 2 days in the presence of 20ng/ml IL-4 plus 20ng/ml IL-13 or the above spent cell culture medium. An FR-positive subclone of the murine macrophage-derived RAW264.7 cell line was obtained as previously described (19). Cells were then analyzed for FRβ expression by flow cytometry (see **Supplemental Tables S1**, **S2**).

To assess the effect of TLR7-1A and FA-TLR7-1A on cytokine production, FR-positive Raw264.7 cells were seeded to 96-well plate in a density of 0.2 million cells/well. Cells were then incubated for 24 hours with indicated concentration of either FA-TLR7-1A or TLR7-1A in the absence or presence of 100X excess folate-glucosamine (competition). The culture medium was then collected for cytokine analysis using ELISA.Analysis of TLR7-1A and FA-TLR7-1A induced cytokine release in fresh whole human blood was performed similarly, except the whole blood was treated with either a vehicle control, 100nM TLR7-1A, or 100nM FA-TLR7-1A for 4 hours before centrifugation for 10 minutes at 1,000g. The resulting cell pellets were hemolyzed in RBC lysis buffer (BioLegend; Cat# 420301) and the residual white cells were analyzed using quantitative polymerase chain reaction (qPCR) to quantitate cytokine mRNA levels. Plasma supernatants were also analyzed by ELISA for secreted cytokines as described below.

### Animal husbandry

Six- to eight-week-old female Balb/c mice were purchased from Envigo and used for all live animal studies. Mice were housed in accordance with protocols approved by Purdue University Animal Care and Use Committee. Water and folate-deficient chow (Envigo, Cat#TD.00434) were freely available.

### Analysis of FRβ and TLR7 expression in murine tumors

Mice were subcutaneously injected with 4T1 cells (50,000 cells/mouse) and tumor sizes were measured every other day. When tumors reached 500-600mm^3^, mice were sacrificed and perfused intracardially with chilled phosphate buffered saline (PBS). Part of each tumor was then dissociated with tumor dissociation kit (Miltenyi, Cat#130-096-730) and erythrocytes were depleted using RBC lysis buffer. After two washes with cold PBS, the resulting single cell suspensions were analyzed by flow cytometry for the desired phenotypic markers (**Supplemental Tables S1**, **S2**). The remaining portion of each tumor was fixed in 4% formalin, followed by cryoprotection in 30% sucrose solution, then cryosectioned into 15μm sections and stored as free-floating sections at 4°C for subsequent immunofluorescence staining.

### Analysis of total and functional FRβ expression in human PBMCs, murine PBMCs, spleens and Raw264.7 cells

For analysis of monocytes from the peripheral blood of healthy mice, peripheral blood was collected by cardiac puncture prior to washing 3x in PBS. Spleens from the same mice were also resected and gently minced before extrusion through a 70 μm cell strainer. Erythrocytes in the resulting cell suspension were then lysed using RBC lysis buffer and residual cells were washed 2x in PBS. The resulting PBMCs and splenocytes were then stained with the desired antibodies and analyzed by flow cytometry (**Supplementary Tables 1**, **2**). Analysis of total and functional FRβ expression in human PBMCs was performed similarly, except that the PBMCs were isolated from healthy human peripheral blood as described above.

### Evaluation of cytokine induction following systemic administration of TLR7-1A and FA-TLR7-1A to healthy mice

Healthy mice were injected intravenously with 10nmol/mouse of either FA-TLR7-1A or TLR7-1A, and peripheral blood was collected at the indicated timepoints. Blood was then centrifuged at 4°C and 1,000g for 10 minutes, and the resulting plasma was analyzed for cytokines using ELISA as described below.

### Flow cytometry

Single cell suspensions from both mice and human samples were first stained with Zombie Violet (BioLegend, Cat#423114) and then washed 2x with PBS prior to incubation with anti-mouse TruStain FcX™ (BioLegend, Cat#101319) or anti-human TruStain FcX™ (BioLegend, Cat# 422301), respectively, to block nonspecific Fc domain binding. Cells were then stained with the desired antibodies listed in **Supplementary Tables 1**, **2** and washed 2x with PBS. For intracellular TLR7 staining, cells were fixed and permeabilized using Cyto-Fast™ Fix/Perm Buffer Set (BioLegend, Cat#426803), followed by staining with the anti-mouse TLR7 monoclonal antibody (**Supplementary Tables 1**, **2**). To quantitate the functional subset of FRβ expressing cells, a folate receptor-targeted fluorescent dye conjugate (folate-Cy5, synthesized in-house; or folate-fluorescein, MedChemExpress, #910661-33-5) were used. After washing, cells were resuspended in FACS buffer and examined using an Attune NxT flow cytometry prior to data analysis using Attune Cytometric Software or FlowJoucodep™ v10.

### ELISA analysis of cytokines in plasma and cell culture supernatant

Human IL-6, TNFα and IFNγ were quantified using an ELISA MAX™ Deluxe Set (BioLegend) kit, while similar mouse-specific kits (BioLegend) were used for quantitation of mouse IL-6 and TNFα.

### Immunofluorescence staining

4T1 tumors were collected, processed, and stained as described previously, with minor modifications (20). Briefly, sections were immunostained overnight with anti-F4/80 (BioLegend, Cat#123101, 1:1000), anti-TLR7 (Novus Biologicals, Cat#NBP2-27332SS, 1:1500), and anti-FRβ (AF647, F3 IgG29, 1:100) (21) primary antibodies. Secondary antibody staining was performed with Alexa Fluor^®^ 488 and Alexa Fluor^®^ 594 (Jackson ImmunoResearch, 1:2000) antibodies. Sections were then mounted on glass slides in ProLong Gold Antifade Mounting media. Prepared slides were shipped to iHisto, Inc. for whole slide scanning.

### qPCR analysis of RNA expression

After erythrocyte depletion, RNA was isolated from total leukocytes, reverse-transcribed to cDNA and analyzed by qPCR using the methods and primer sequences described previously (18). Melting curve analysis was performed to validate specificity. Each sample was analyzed in triplicate for each marker.

### Single cell RNA-seq sample preparation and library construction

The 4T1 tumor model and treatment strategy described in our previous publication were used (17). In brief, 3nmol/mouse of FA-TLR7-1A was administered intravenously 5 times per week for two weeks. Mice were then sacrificed and tumors were dissociated as described above to obtain single cell suspensions. Viable cells were enriched using Dead Cell Removal Microbeads (Miltenyi, Cat# 130-090-101) and an LS Colum (Miltenyi, Cat# 130-042-401) in the magnetic field of a MidiMACS™ Separator, after which cells were diluted to 1 million cells/ml in ice-cold PBS containing 0.04% Ultrapure BSA (50mg/ml, ThermoFisher, Cat#AM2616) and cell viability (>95%) was again validated by flow cytometry. The whole procedure was completed within 1 hour and the resulting viable cells were used for scRNA-seq library construction immediately.

Samples were handled in accordance with 10x Genomics protocols (Next GEM Single Cell 3’ Reagent Kit v3.1). Following 10x loading suggestions, ~16,000 cells were loaded into each well of the chip for a targeted cell recovery of 10,000 cells per lane. Chip G from 10x Genomics was utilized for GEM generation and all steps following GEM generation were carried out as described in the 10x Genomics manual. After GEM processing on the chromium controller, all steps to amplify cDNA and generate libraries were carried out according to the 10x Genomics manual. Quality control procedures were performed by the Purdue University Genomics Core Facility after cDNA amplification and library generation. All sequencing was then performed by the Purdue University Genomics Core Facility. Sequencing was targeted to approximately 50,000 paired end reads per cell.

### Analysis of single cell RNA-seq data

Sequencing reads from the Chromium system were de-multiplexed and processed using the CellRanger pipeline v6.0.2 (10x Genomics). CellRanger count was then used for alignment, filtering, barcode counting, and unique molecular identifier (UMI) counting. All reads were aligned to the ENSEMBL mouse genome version mm10 using the STAR aligner v2.7.2a (22). CellRanger was run with the number of expected cells set to 5,000. R version 4.0.1 and Bioconductor version 3.11 were used in all statistical analyses. Cells that had fewer than 200 or greater than 6000 observed genes were discarded. Cells were also removed if greater than 15% of all reads mapped to mitochondrial genes or greater than 50% of all reads mapped to ribosomal genes. The metrics indicate high quality data and that the average number of cells captured approached the intended number of 5000 cells.

All scRNA-seq data were deposited to GEO and are available under accession number GSE236443. Seurat version 4.0.5 (23) was used for data normalization and cell clustering based on differential gene expressions. Data were normalized using scTransform v0.3.2 (24) followed by integration using standard Seurat workflow. These “corrected” data, after permutation and selection of the first 20 principal components based on principal component analysis (PCA) scores, were used for downstream analysis. Unsupervised clustering was performed in Seurat, which uses graph-based approaches to first construct K-nearest neighbor graphs (K = 20) and then identifies clusters by iteratively forming communities of cells to optimize the modularity function. The number of clusters were determined using the Louvain algorithm (25) for community detection (as implemented in Seurat), with a resolution of 0.6. The correct resolution to use was determined both visually through plots and heat maps as well as using clustering trees via the Clustree R package v0.4.3 (26), selecting a resolution that provides stable clusters. Biomarkers were considered statistically significant at a 5% false discovery rate (FDR) using the Wilcoxon rank sum test (27). Differentially expressed genes between sample groups were identified using the Wilcoxon rank sum test with an FDR cutoff of 5%. P-values were corrected for multiple testing using the Benjamini-Hochberg method (28) wherever applicable. Sub-clustering of major cell-types (myeloid, T/NK, and fibroblasts), optimal clustering, biomarker identification and differential expression analysis were performed using the same approach as above. Quantitation of the changes in population sizes of the different cell clusters was performed using the permutation test in R-package scProportionTest (29). Changes in population sizes with FDR < 0.05 were denoted as statistically significant.

Further analysis of the correlation between FRβ expression and cancer patient survival was done by extracting FRβ expression and patient survival data from the TCGA database and analyzed using UALCAN (30).

### Characterization of single cell RNA-seq clusters

All cells from a tumor mass were first clustered into a total of 19 primary clusters. To identify the cells within these clusters, differentially expressed marker genes specific to each cluster were analyzed. The clusters were identified and labeled accordingly, as provided in **Supplementary Table 3**. To further investigate the impact of FA-TLR7-1A treatment on myeloid cells, T cells/NK cells and fibroblasts, these three major cell groups were extracted separately and subjected to sub-clustering as described above. The differentially expressed marker genes, as listed in **Supplementary Table 3**, were used to characterize the relevant sub-clusters within these cell types.

### Statistical analysis

Statistical analyses were conducted using GraphPad Prism version 10 software (Graphpad; San Diego, CA). All figures present mean ± s.d. values unless stated otherwise. To compare multiple groups, a one-way ANOVA followed by a Dunnett’s multiple comparison test was utilized when applicable. Significance levels were marked as follows: (*P < 0.05, **P < 0.01, ***P < 0.001, ****P < 0.0001).

When analyzing scRNA-seq data, we identified statistically significant changes between sample groups in population size and differentially expressed genes by utilizing an FDR threshold of < 0.05, as mentioned above.

## Results

### Analysis of folate receptor beta expression and functionality in healthy and malignant tissues

**Figure 1:**
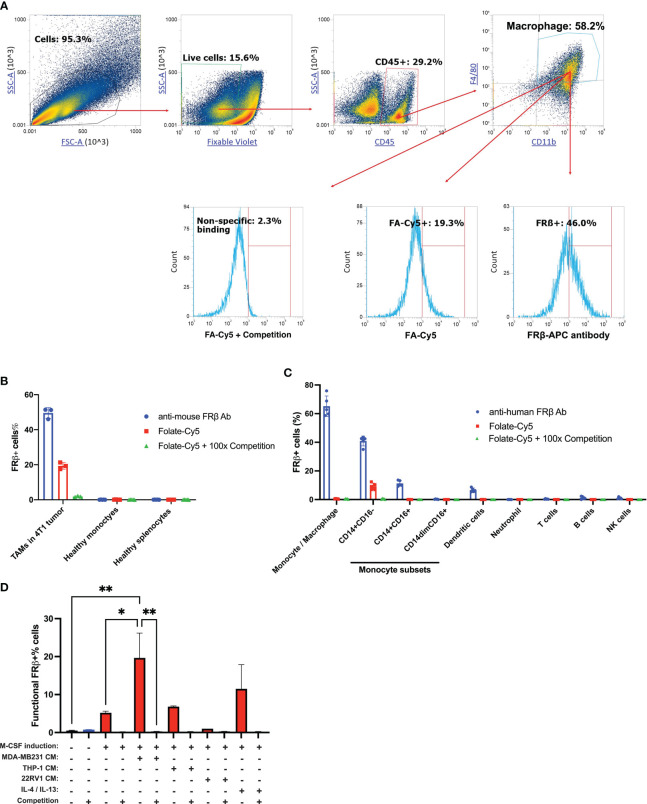
Analysis of FRβ expression and induction of its folate binding ability in myeloid cells from murine and human blood/tissue samples. **(A)** 4T1 tumors were dissociated as described in Methods and stained for live cells (zombie violet) followed by white cells (CD45+), and then macrophages (anti-F4/80 and anti-CD11b). To determine the fraction of FRβ-expressing cells that can bind folic acid, anti-F4/80 and anti-CD11b positive cells were treated with either anti-FRβ monoclonal antibody (46% positive) or a folate-linked fluorescent dye (FA-Cy5) in the presence (0.3% positive) or absence (13% positive) of excess folate-glucosamine to block all functional folate receptors. **(B)** Analysis of the percent of myeloid cells that express FRβ or bind folic acid (folate-Cy5) in 4T1 tumors (left bars), peripheral blood monocytes (central bars), and splenocytes (right bars) from tumor-bearing (n=3) and healthy mice (n=3), as indicated. **(C)** Analysis of FRβ expression (using anti- FRβ monoclonal antibody) and folate (folate-Cy5) binding ability in healthy human PBMCs (n=5). The percent of cells staining positive for FRβ protein (blue), functional FRβ (red), and nonspecific binding in the presence of 100-fold excess folate-glucosamine (green) are shown. **(D)** Analysis of the percentage of healthy human monocytes that can bind folic acid (folate-fluorescein) after culturing for 7 days in M-CSF to mature the monocytes to macrophages and then incubating them for 48 hours in the indicated cancer cell conditioned medium or cytokines (IL-4 + IL-13). To assess FRβ-specific binding, each preparation was also incubated in the presence of 100-fold excess folate-glucosamine (competition) to block all FRβ binding sites. Mean ± SD. Statistical significance between groups was determined using one-way ANOVA (*P<0.05, **P<0.01).

As noted in the Introduction, because FRβ is expressed solely on myeloid cells (5, 6) and since its expression in tumor tissues correlates with poor overall survival (5, 30–32) (**Supplementary Figure 1**), FRβ has become both an important marker for tumor infiltrating TAMs/MDSCs and an intriguing target for receptor-directed therapeutics (4, 6, 7, 9). However, a major question has remained regarding the ability of FRβ to bind folic acid, since some publications report high affinity binding (6, 33, 34) while others report no detectable binding (35, 36). To evaluate whether the FRβ-expressing myeloid cells in a tumor mass might bind folic acid, 4T1 murine breast cancer cells were implanted in immune competent Balb/c mice and resulting tumors were dissociated into component cells prior to incubation with a folic acid conjugate of the fluorescent dye Cy5 (FA-Cy5). As shown in **Figure 1A**, although anti-FRβ monoclonal antibody stained ~46% of CD11b+F4/80+ cells, FA-Cy5 bound to only 19% of the same TAMs; i.e. suggesting that not quite half of all FRβ+CD11b+F4/80+ cells express a functional folate receptor. Moreover, because FA-Cy5 binding could be blocked by excess folate-linked glucosamine (i.e. a competitor of folate binding to FR), we conclude that FA-Cy5 binding was FRβ-specific. These data demonstrate that less than half of TAMs in the tumor mass express a functional FRβ.

To examine FRβ expression and functionality in nonmalignant tissues, freshly drawn blood from both tumor-bearing and nontumor-bearing mice was incubated with either anti-FRβ antibody or FA-Cy5, and the above flow cytometry experiments were repeated. As shown in **Figure 1B** and **Supplementary Figure 2**, fewer than 1% of circulating monocytes from the blood of healthy mice bound anti-FRβ monoclonal antibody, and essentially none of the monocytes from the same mice were stained with FA-Cy5. Because similar results were also obtained on myeloid cells from the spleens of healthy mice (**Figure 1B**), we conclude that functional FRβ is primarily detected in a tumor mass.

To assess the expression and functionality of FRβ on human peripheral blood mononuclear cells (PBMCs), we isolated fresh PBMCs from healthy donors and incubated them with both a monoclonal antibody to human FRβ and FA-Cy5, approximately as described above. In contrast to murine monocytes, ~40% of human monocytes stained positive for FRβ (i.e. suggesting that the receptor is present on a population of human monocytes). However, only a fraction of these monocytes bound the fluorescent folate conjugate, i.e. indicating that the folate receptors were largely nonfunctional (**Figure 1C** and **Supplementary Figures 3A–H**).

### Identification of stimuli that convert nonfunctional to functional folate receptor beta

Because previous studies had suggested that myeloid cells could bind folic acid and its conjugates in solid tumors, we then decided to investigate the nature of the tumor stimuli that might induce folate binding by FRβ. For this purpose, we collected conditioned cell culture media from three different cancer cell lines and examined their abilities to activate FRβ in human PBMC-derived monocytes. As shown in **Figure 1D**, while 22RV1 human prostate cancer cell medium induced little or no FRβ activation, THP1 human monocytic leukemia culture medium promoted moderate FRβ activation and MDA-MB-231 human breast cancer medium stimulated strong FRβ activation. Importantly, incubation of macrophages from the same healthy donors in the presence of IL-4/IL-13 cytokine combination [commonly used to differentiate human monocytes into M2-like immunosuppressive macrophages (18, 37)] also induced FRβ activation. These data demonstrate that conversion of FRβ from nonfunctional to functional state can derive from factors secreted by cancer cells or induced by a combination of the immunosuppressive cytokines. The fact that these same inducing factors are largely absent from healthy tissues further explains why macrophages with functional FRβ are essentially absent from noninflamed tissues (17, 33).

### Identification of cells that co-express FRβ and TLR7

**Figure 2:**
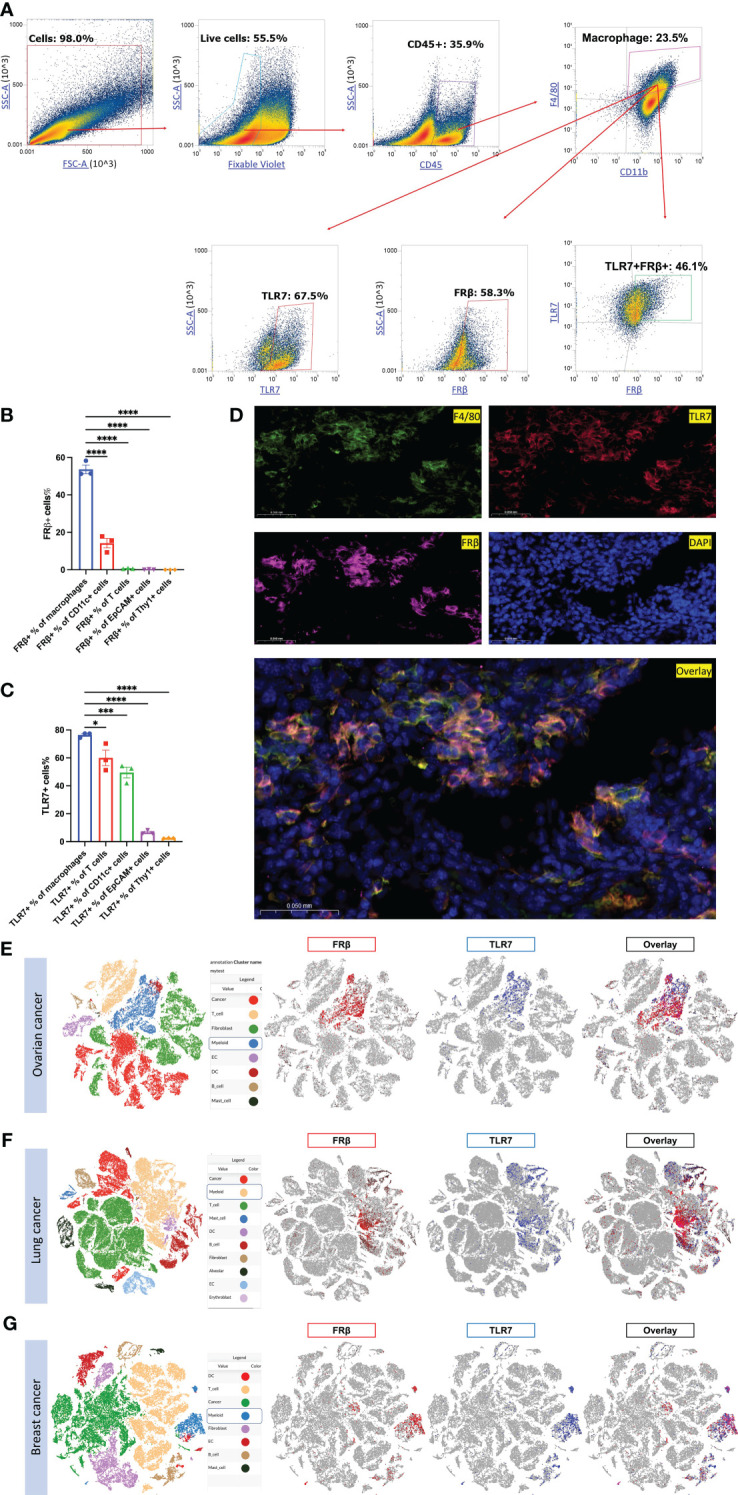
Evaluation of FRβ and TLR7 expression and co-expression in murine and human cancers. **(A)** 4T1 tumors (n=3) dissociated as described in Methods and stained for live cells (zombie violet) followed by white cells (CD45+), macrophages (anti-F4/80 and anti-CD11b), and then TLR7 and FRβ. **(B)** Except for a fraction of myeloid CD11c+ dendritic cells, only TAMs and MDSCs expressed FRβ. **(C)** In contrast, many cell types expressed TLR7. **(D)** Immunofluorescent staining shows that FRβ and TLR7 co-localize with F4/80 staining in tumor sections. Single cell RNA seq data collected on multiple samples of human ovarian **(E)**, lung **(F)** and breast **(G)** cancers published elsewhere (38) were interrogated for FRβ and TLR7 expression. Cell cluster identities are shown on the left and expression of FRβ (red) and TLR7 (blue) in these cell clusters are shown on the right. FRβ and TLR7 are only co-expressed on myeloid cells in both cancer types. Mean ± SEM are shown for **(B, C)** Statistical significance between groups was determined using one-way ANOVA (*P < 0.05, ***P < 0.001, ****P < 0.0001).

Motivated by the fact that functional FRβ is almost exclusively expressed in malignant (or inflamed) tissues, we next undertook to determine which immune-activating pathway might be prominent in FRβ+ TAMs/MDSCs, but only activatable by intracellular drugs that could be internalized by FRβ-mediated endocytosis. To determine whether an intracellular receptor like toll-like receptor 7 (TLR7) might co-localize with FRβ in TAMs or MDSCs, 4T1 tumors from the aforementioned mice were dissociated into single cell suspensions and analyzed for both FRβ and TLR7. As shown in **Figures 2A, B** and **Supplementary Figures 4A–E**, 4T1 cancer cells themselves expressed no folate receptors, however, ~58% of TAMs/MDSCs and ~14.2% of myeloid-derived dendritic cells expressed FRβ. Moreover, examination of the same cell suspensions for TLR7 revealed that ~67.5% of TAMs/MDSCs expressed TLR7, and ~46.1% of these same cells produced both FRβ and TLR7 (**Figure 2A**). Although TLR7 was also widely expressed in other cell types, including T cells, dendritic cells, and a fraction of cancer cells (**Figure 2C**, **Supplementary Figures 4B–D**), the only cells that expressed both FRβ and TLR7 resided in the subset of myeloid cells. Not surprisingly, immunofluorescent staining of the same tumor tissues with antibodies to FRβ and TLR7 confirmed that FRβ and TLR7 colocalize only in tumor macrophages (**Figure 2D**). Because a similar colocalization was also found by scRNA-seq in human ovarian, lung and breast cancer specimens (**Figures 2E–G**), we conclude that FRβ is commonly co-expressed in M2-like macrophages with TLR7 receptors (**Supplementary Figures 6A–C**) (4, 6, 7, 38). Because TLR7 receptors are present in intracellular endosomes (39), and since FRβ traffics to intracellular endosomes (40, 41), this colocalization suggests that a folate-targeted TLR7 agonist might activate TAMs/MDSCs in solid tumors without stimulating similar myeloid cells in healthy tissues.

### Specificity of an FRβ-targeted immune stimulant for FRβ-expressing macrophages

**Figure 3:**
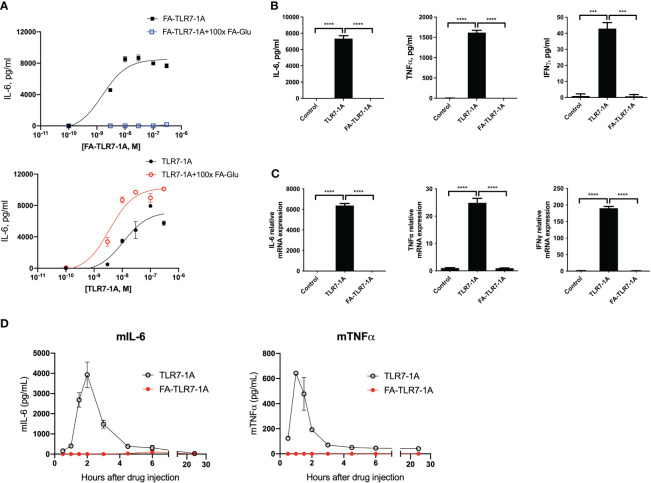
Comparison of cytokine induction by nontargeted TLR7-1A and FA-TLR7-1A. **(A)** An FR-positive subclone of the murine macrophage-derived RAW264.7 cell line were treated with varied concentrations of either FA-TLR7-1A or TLR7-1A in the absence or presence of 100x excess folate-glucosamine (FA-Glu, competition) for 24 hours, after which the culture medium was quantitated for IL-6 production. **(B)** IL-6, TNFα, and IFNγ were quantitated in anticoagulated fresh healthy human whole blood by ELISA four hours following treatment with 100 nM TLR7-1A, 100 nM FA-TLR7-1A or vehicle control. **(C)** mRNAs for IL-6, TNFα, and IFNγ were quantitated in the white cell fractions of the same whole blood samples. **(D)** Healthy mice were injected intravenously with 10 nmoles of either TLR7-1A or FA-TLR7-1A, after which IL-6 and TNFα levels were quantitated in derived blood samples as a function of time. Mean ± SD. Statistical significance between groups was determined using one-way ANOVA (***P < 0.001, ****P < 0.0001). vehicle control= 0.2% DMSO in medium.

With acquisition of functionality in FRβ restricted to the tumor microenvironment, we decided to evaluate whether a folate-targeted TLR7 agonist might be able to induce a proinflammatory phenotype in tumor myeloid cells without activating immune cells in healthy tissues. For this purpose, we linked folic acid (FA) to a potent TLR7 agonist (TLR7-1A) in a manner that created a conjugate (FA-TLR7-1A) that would be internalized by functional FRβ-expressing cells but impermeable to cells lacking a functional folate receptor (17). To test the potency and specificity of this conjugate in stimulating FRβ+ myeloid cells, we treated an FRβ-expressing clone of murine macrophage-derived RAW264.7 cells (**Supplementary Figure 5**) with either FA-TLR7-1A or nontargeted TLR7-1A in the absence or presence of 100-fold excess folate-glucosamine (FA-Glu) to block all FRβ. As shown in **Figure 3A**, both FA-TLR7-1A and TLR7-1A induced production of IL-6 with similar potency in the absence of FA-Glu, but only nontargeted TLR7-1A stimulated IL-6 release in the presence of FA-Glu. These observations demonstrate that FA-TLR7-1A can only induce IL-6 production in functional FRβ-expressing macrophages, and that FA-TLR7-1A is inactive when FRβ-mediated uptake is blocked by excess FA-Glu.

Because the ability of FA-TLR7-1A to stimulate proinflammatory cytokine production in a tumor microenvironment without inducing cytokine release systemically could be therapeutically useful, we elected to confirm this selectivity in humans by incubating fresh whole human blood from healthy donors with either FA-TLR7-1A or free TLR7-1A and measuring secretion of inflammatory cytokines (**Figures 3B, C**). Although free TLR7-1A was found to induce strong TNFα, IL-6, and IFNγ production, FA-TLR7-1A elicited no detectable cytokines in the same samples, i.e. because healthy human PBMCs express no functional folate receptors (**Figure 1C**) and FA-TLR7-1A cannot enter FR-negative cells.

Finally, to examine this same dependence on functional FRβ in live mice, healthy mice were intravenously injected with either free TLR7-1A or FA-TLR7-1A, and serum cytokine levels were measured in the peripheral blood as a function of time. As shown in **Figure 3D**, although TLR7-1A promoted strong systemic cytokine release, similar injection of FA-TLR7-1A promoted no cytokine discharge. Considered together with the data in **Figures 3A–C**, these results establish that FA-TLR7-1A will only induce inflammatory cytokine production in cells that express functional FRβ, and because FRβ is only activated in tumor (or inflamed) tissues, these data argue that FA-TLR7-1A should constitute a safe method for reprogramming TAMs and MDSCs in a tumor mass without inducing their toxic cytokine release in healthy tissues. This specificity is obviously important because systemic activation of the immune system has prevented the FDA from approving any systemically administered TLR7 agonists in the past (42–44).

### Analysis of genes induced by FA-TLR7-1A in FRβ-expressing cells by single cell RNA-seq

**Figure 4:**
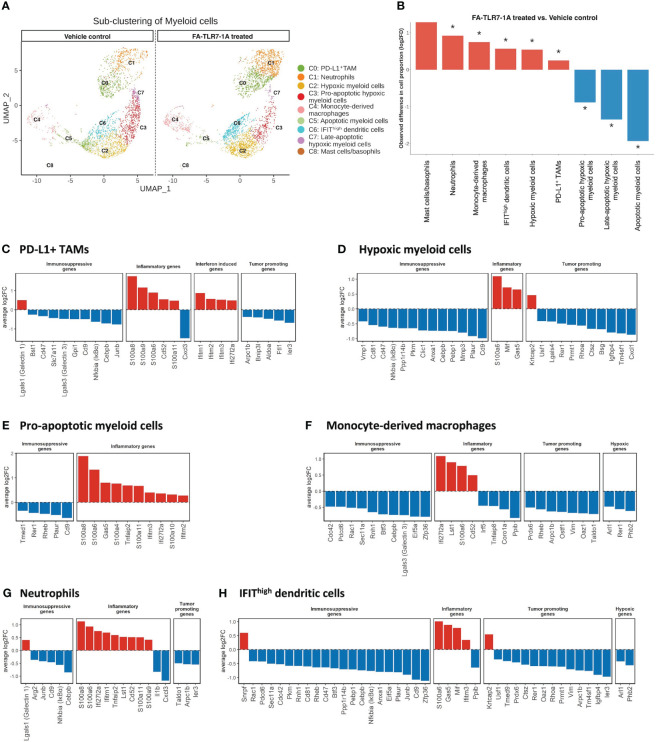
Analysis of the effect of FA-TLR7-1A on myeloid cells. Mice bearing 4T1 tumors were treated with either FA-TLR7-1A or vehicle control, and their tumor cells were analyzed by single cell RNA-seq. Infiltrating tumor myeloid cells were sub-clustered based on gene expression patterns (see Methods) into 9 populations (panel **A**) and changes in their population sizes were quantitated (panel **B**). Statistically significant (FDR < 0.05) changes in population sizes are denoted with *. Changes in relevant gene categories were also quantitated for the indicated myeloid cell subpopulations **(C–H)**. All genes shown in **(C–H)** are statistically significant (FDR < 0.05). vehicle control = 3% DMSO in PBS.

With the mechanism of FA-TLR7-1A’s tumor specificity established, the question finally arose regarding which genes in TAMs and MDSCs might be activated by FA-TLR7-1A. To explore this question, we employed single cell RNA sequencing (scRNA-seq) to compare the gene expression profiles of the major tumor-infiltrating myeloid cell subtypes following intravenous administration of FA-TLR7-1A. 4T1 tumor bearing mice were treated with either FA-TLR7-1A or vehicle control for 2 weeks prior to sacrificing for scRNA-seq analysis (**Supplementary Figure 7**). Tumor cells were first clustered into 19 cell types (**Supplementary Figures 8A–C**), and myeloid cells, T cells/NK cells, and fibroblasts were further sub-clustered for following analysis (**Supplementary Figures 9A–F**). As shown in **Figures 4A, B**, tail vein injection of FA-TLR7-1A promoted significant changes in the cellular composition of the 4T1 tumors, with large increases in the abundances of mast cells, basophils, and neutrophils and smaller rises in the abundances of monocyte-derived macrophages and IFIT^high^ dendritic cells. In contrast, pro-apoptotic hypoxic myeloid cells and fully apoptotic cells were found to significantly decrease in numbers.

Analyses of the FA-TLR7-1A induced changes in myeloid cell gene expression revealed that transcripts of immune-activating and pro-inflammatory genes largely increased in abundance, while immunosuppressive, anti-inflammatory, and tumorigenesis-related transcripts predominantly decreased (**Figures 4C–H**). For example, S100 family genes (S100a8, S100a9, and S100a6) that enhance activation of NLRP3 inflammasomes and in turn promote expression of IL-1β and IL-18 are seen to increase in myeloid subpopulations (45) whereas immunosuppressive genes [Cd47 (46), Cd9 (47), Lgals3 (48), and Junb (49) etc.), tumorigenesis genes (Arpc1b (50), Ier3 (51), and Cxcl1 (52), etc.) and hypoxic genes (Phb2 (53), Rer1 (54), and Arl1 (55)] are observed to consistently downregulate. Not surprisingly, signaling components downstream of the NFκB (e.g. Ifitm1, Ifitm2, Ifitm3 and Ifi2712a) (56) are also found to increase in the TAMs/MDSCs of FA-TLR7-1A treated mice. Taken together, these data argue that FA-TLR7-1A shifts the tumor myeloid population towards a more pro-inflammatory, anti-tumor phenotype.

**Figure 5:**
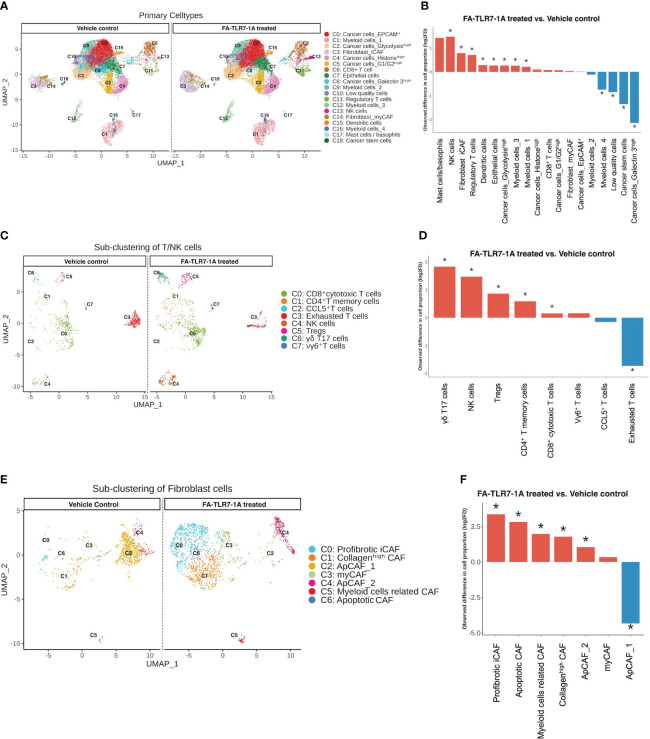
Analysis of the effect of FA-TLR7-1A on non-myeloid cells in tumor masses. Total cells digested from the 4T1 tumors in **Figure 4** were segregated into 19 clusters. T cells/NK cells were further sub-clustered to 8 subtypes, while fibroblasts were sub-clustered to 7 subtypes. All clustering was unsupervised. **(A)** Uniform Manifold Approximation and Projection (UMAP) of all cell clusters between FA-TLR7-1A and vehicle control treated mice. **(B)** Quantitation of the changes in population sizes of the different cell clusters. **(C)** Comparison of the UMAP of the T cell/NK cell subpopulations. **(D)** Quantitation of the changes in population sizes of the cell clusters in panel **(C, E)** Comparison of the UMAP of the fibroblast subpopulations. **(F)** Quantitation of the changes in population sizes of the different cell clusters in panel **(E)** vehicle control = 3% DMSO in PBS. Statistically significant (FDR < 0.05) changes in population sizes are denoted with *.

Because myeloid cells secrete cytokines and other factors that can influence the abundances and gene expression profiles of other cells in the same TME, we finally examined the changes in numbers of non-myeloid cell types in the same tumor masses. As shown in **Figures 5A, B**, cancer cells, endothelial cells, apoptotic cells and immunosuppressive myeloid cells generally decreased in number, while T/NK cells, fibroblasts, and mast cells increased in abundance, i.e. confirming the general shift towards a more immune-activating TME. Among the T and NK cell populations that were impacted most by FA-TLR7-1A, the exhausted T cells appeared to decline most dramatically, while the γδT17 cells, NK cells, Tregs and memory T cells all increased in number (**Figures 5C, D**). Not surprisingly, the abundance of profibrotic inflammatory cancer associated fibroblasts (iCAF) and collagen^high^ CAFs also increased, while the antigen-presenting CAF (ApCAF) population showed mixed trends. We speculate that this latter incongruity may have derived from initiation of a tissue repair response that was triggered by the inflammation induced by FA-TLR7-1A.

## Discussion

The purpose of these studies was to explore why myeloid cells in a tumor microenvironment could be reprogrammed by FRβ-targeted drugs, but myeloid cells from healthy tissues in the same mice could not. Our data reveal that FRβ, the targeted receptor, is essentially absent from healthy tissues in live mice, but expressed predominantly in nonfunctional form on many circulating monocytes and macrophages in healthy humans. In contrast, FRβ+ was found to be abundant on both murine and human macrophages in solid tumors, and ~40% of these receptors in the murine tumor microenvironment were shown to be functionally active. That a similar nonfunctional to functional transition likely occurs upon infiltration of FRβ+ monocytes/macrophages into human tumors could be established by demonstrating that FRβ on cultured human monocyte-derived macrophages acquires folate binding ability upon exposure to factors secreted by human cancer cells. Indeed, human tumors that only express the beta isoform of the folate receptor can be readily imaged with folate-targeted fluorescent dyes in human lung cancer patients, i.e. demonstrating that their FRβ are functional (57). While the nature of the change in FRβ from nonfunctional to functional state could not be ascertained by sensitive mass spectrometry analyses (data not shown), the selective activation of FRβ+ in tumor tissues enables targeting of drugs to myeloid cells in solid tumors without perturbing their properties in healthy tissues. This selectivity may be important for treatment of cancers with immune stimulants, since potent immune activators such as TLR7 agonists have failed in human clinical trials because their systemic activation of the immune system could not be tolerated (42–44).

Although several publications have noted the presence of a nonfunctional FRβ on human monocytes/macrophages (33–36), this is the first paper to report that nonfunctional FRβ can be converted to functional FRβ by incubation in either anti-inflammatory cytokines (i.e. IL4 plus IL13) or cancer cell conditioned media. While the evolutionary driving force for this triggered conversion is unclear, it is conceivable that acquisition of folate binding ability could prove beneficial to the tumor. Thus, activation of FRβ could enhance the uptake of a vitamin (i.e. folic acid) required for growth or proliferation of immunosuppressive TAMs and MDSCs in the tumor microenvironment (31, 58, 59). FRβ expression has also been shown to be essential for TAM/MDSC-mediated suppression of CD8+ T cells (4, 7, 8). Internalization of folate by TAMs/MDSCs has further been observed to be necessary for their elevated biosynthesis and secretion of nucleosides, i.e. a process that enables TAMs and MDSCs to supply adjacent cancer cells with building blocks for rapid cell division (12). Because none of these processes are critical for macrophage function in healthy tissues, FRβ activation may explain why FRβ+ macrophages appear to comprise major components of most solid tumors (60–62).

The fact that targeting of TLR7 agonists to FRβ+ TAMs/MDSCs leads to a significant reduction in tumor growth (17) suggests that FRβ+ TAMs/MDSCs are naturally immunosuppressive. Indeed, scRNA-seq analysis of the impact of FA-TLR7-1A on the TME suggests that FRβ+ macrophages contribute prominently to tumor growth, and treatment of FRβ+ TAMs/MDSCs with a TLR7 agonist shifts them to a more inflammatory phenotype. Thus, administration of FA-TLR7-1A increased the infiltration of immune cells, such as CD8+ cytotoxic T cells, CD4+ T cells, γδT17 cells, NK cells, neutrophils, and dendritic cells and decreased the abundances of exhausted T cells and apoptotic myeloid cells. FA-TLR7-1A was also observed to shift the gene expression patterns of various myeloid subsets from immunosuppressive to immune-activating phenotypes. Taken together, these scRNA-seq analyses suggest that FA-TLR7-1A causes a global shift in the tumor immune system to a more pro-inflammatory phenotype.

Because most toll-like receptors are located on cell surfaces (39), they can be easily activated by extracellular agonists, regardless of whether the agonist is incorporated into a targeted conjugate or not. Thus, a folate-targeted TLR 1, 2, 4, 5 or 6 agonists would likely activate any cell expressing anyone of these receptors (39), resulting in systemic activation of the immune system. In contrast, because TLR7 receptors are located in intracellular endosomes (39), a TLR7 agonist must enter the receptor-expressing cell to stimulate it. To exploit this distinction, we designed our FA-TLR7-1A conjugate to be impermeable to any cell lacking a functional folate receptor but internalized by cells expressing FRβ. As a consequence, whereas nontargeted TLR7 agonist was found to strongly activate human PBMCs to produce inflammatory cytokines, FA-TLR7-1A was not. While the aforementioned TLR agonists with extracellular receptors (i.e. TLR1, 2, 4, 5 or 6) could not have been substituted for TLR7-1A, it will be interesting to learn how other TLR agonists with intracellular receptors perform in similar studies.

## Data availability statement

The datasets presented in this study can be found in online repositories. The names of the repository/repositories and accession number(s) can be found below: GEO under accession number GSE236443.

## Ethics statement

The studies involving humans were approved by Purdue University Human Research Protection Program/Institutional Review Board. The studies were conducted in accordance with the local legislation and institutional requirements. The participants provided their written informed consent to participate in this study. The animal study was approved by Purdue University Animal Care and Use Committee. The study was conducted in accordance with the local legislation and institutional requirements.

## Author contributions

FZ: Conceptualization, Data curation, Formal analysis, Methodology, Writing – original draft, Writing – review & editing, Investigation. BH: Writing – original draft, Writing – review & editing, Conceptualization, Data curation, Formal analysis, Investigation, Methodology. SU: Writing – review & editing, Data curation, Formal analysis, Methodology, Software. WL: Writing – review & editing, Conceptualization, Data curation, Formal analysis, Methodology. GC: Writing – review & editing, Data curation, Formal analysis. SH: Writing – review & editing, Data curation, Formal analysis. SZ: Writing – review & editing, Data curation, Formal analysis. JN: Writing – review & editing, Data curation. RJ: Writing – review & editing, Data curation. BZ: Writing – review & editing, Conceptualization, Formal analysis. ML: Writing – review & editing, Data curation. NL: Writing – review & editing, Formal analysis. MS: Writing – review & editing, Conceptualization. TR: Writing – review & editing, Conceptualization. PL: Conceptualization, Funding acquisition, Project administration, Supervision, Writing – original draft, Writing – review & editing, Formal analysis.
