# Supplementary figures and images for: Tumor-specific activation of folate receptor beta enables reprogramming of immune cells in the tumor microenvironment
Source: Front Immunol. 2024 Feb 7;15:1354735. doi: 10.3389/fimmu.2024.1354735 (PMC10879311; doi:10.3389/fimmu.2024.1354735)

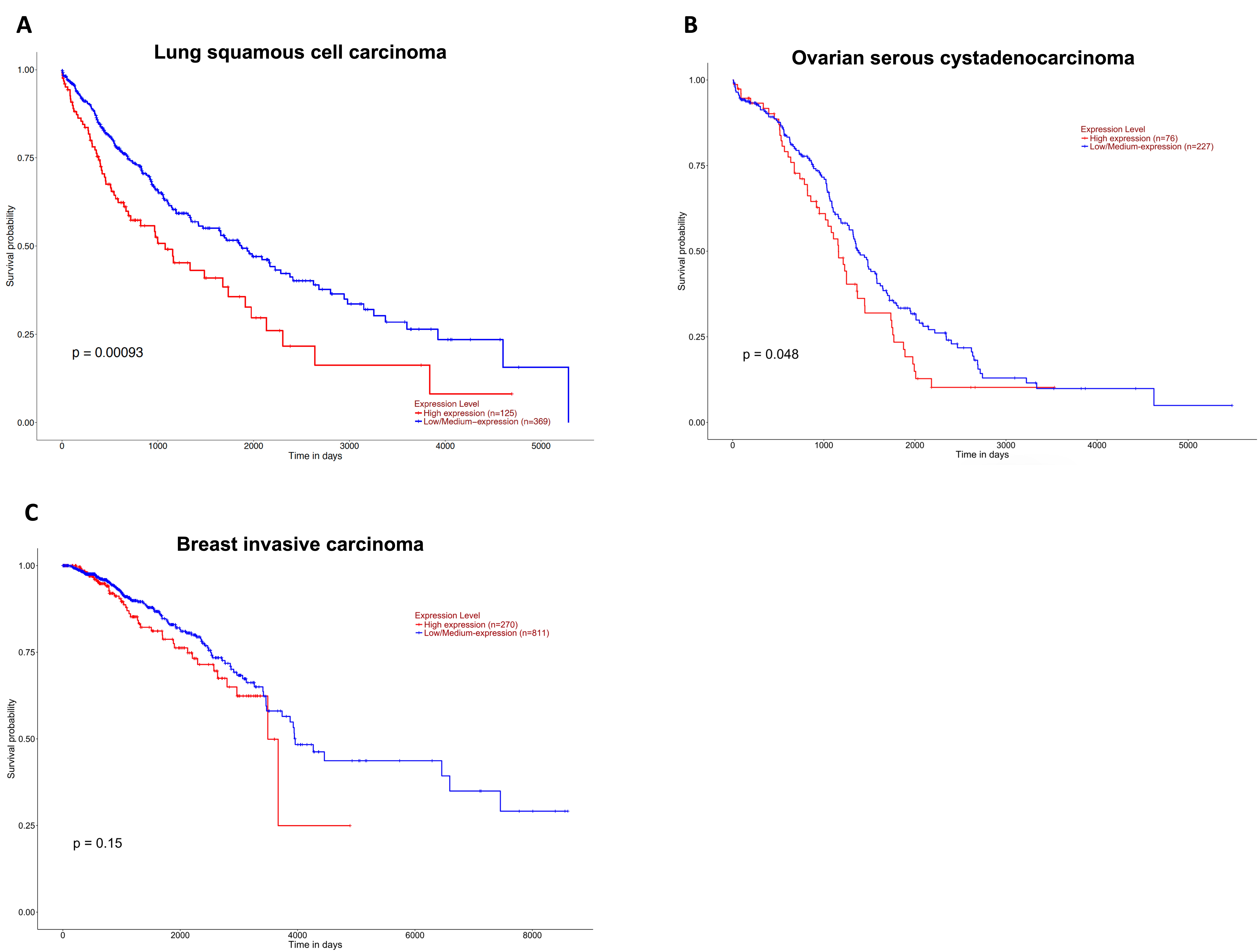

Supplement: Supplementary file 1 [file Image_1.jpeg]

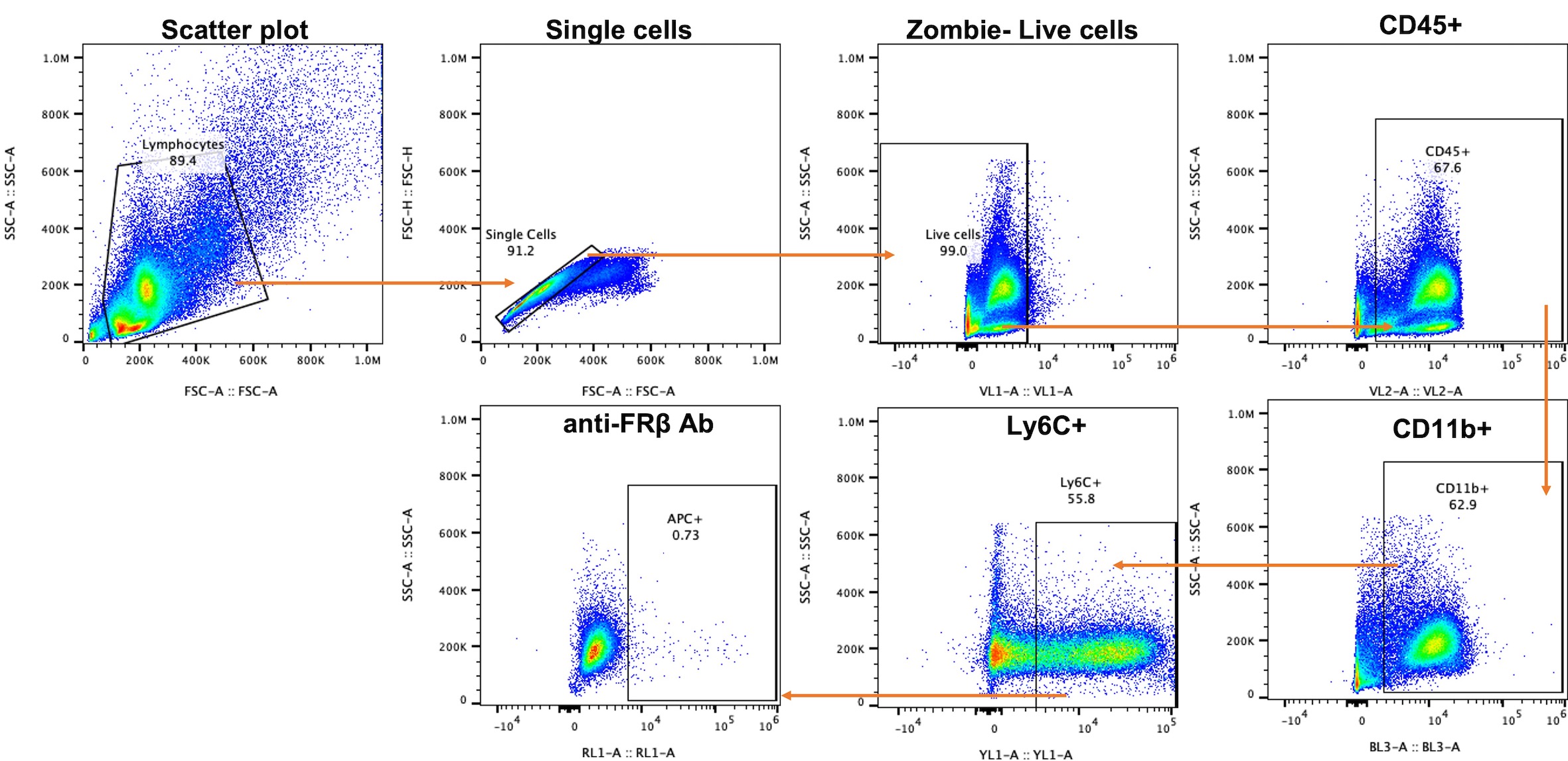

Supplement: Supplementary file 2 [file Image_2.jpeg]

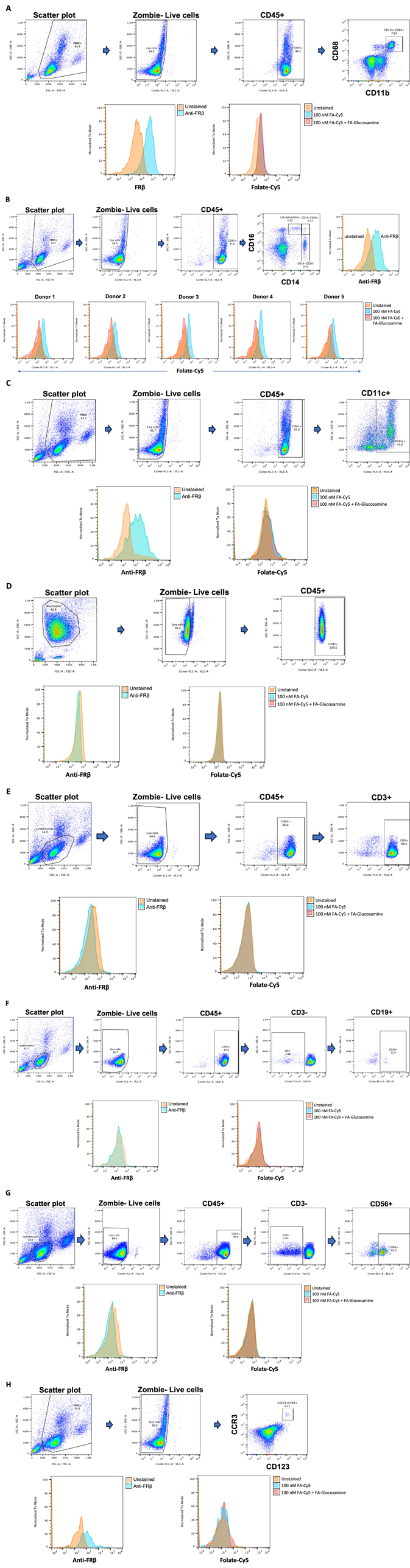

Supplement: Supplementary file 3 [file Image_3.jpeg]

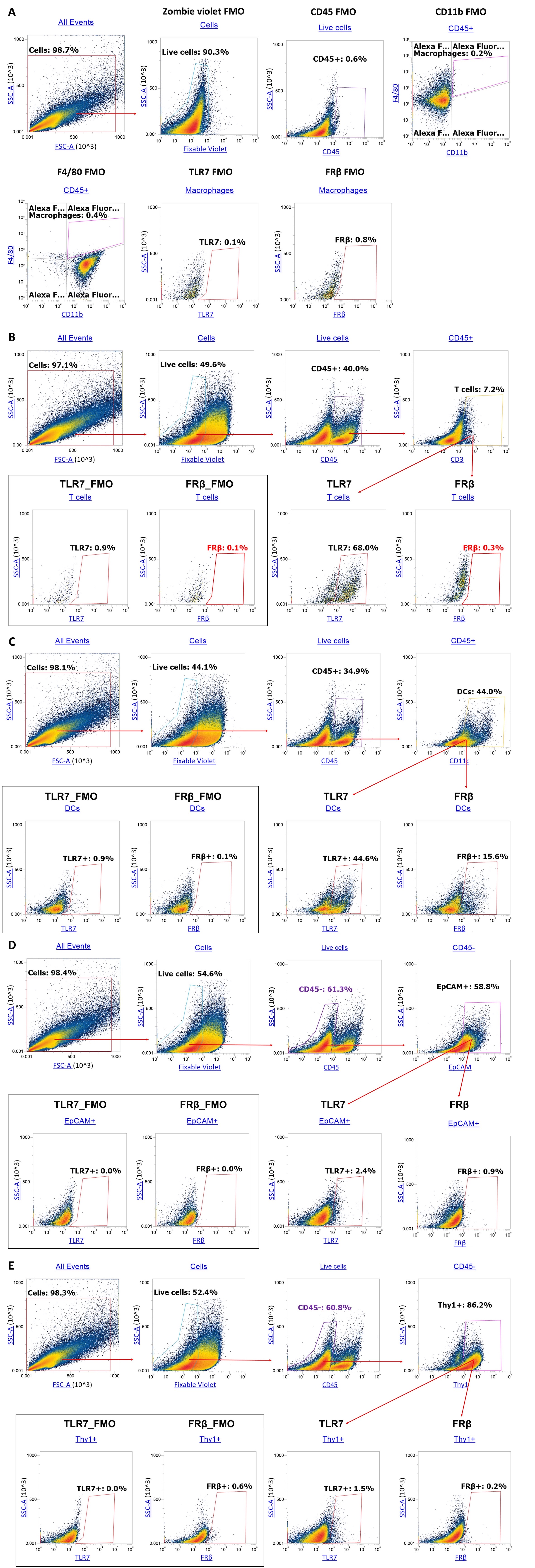

Supplement: Supplementary file 4 [file Image_4.jpeg]

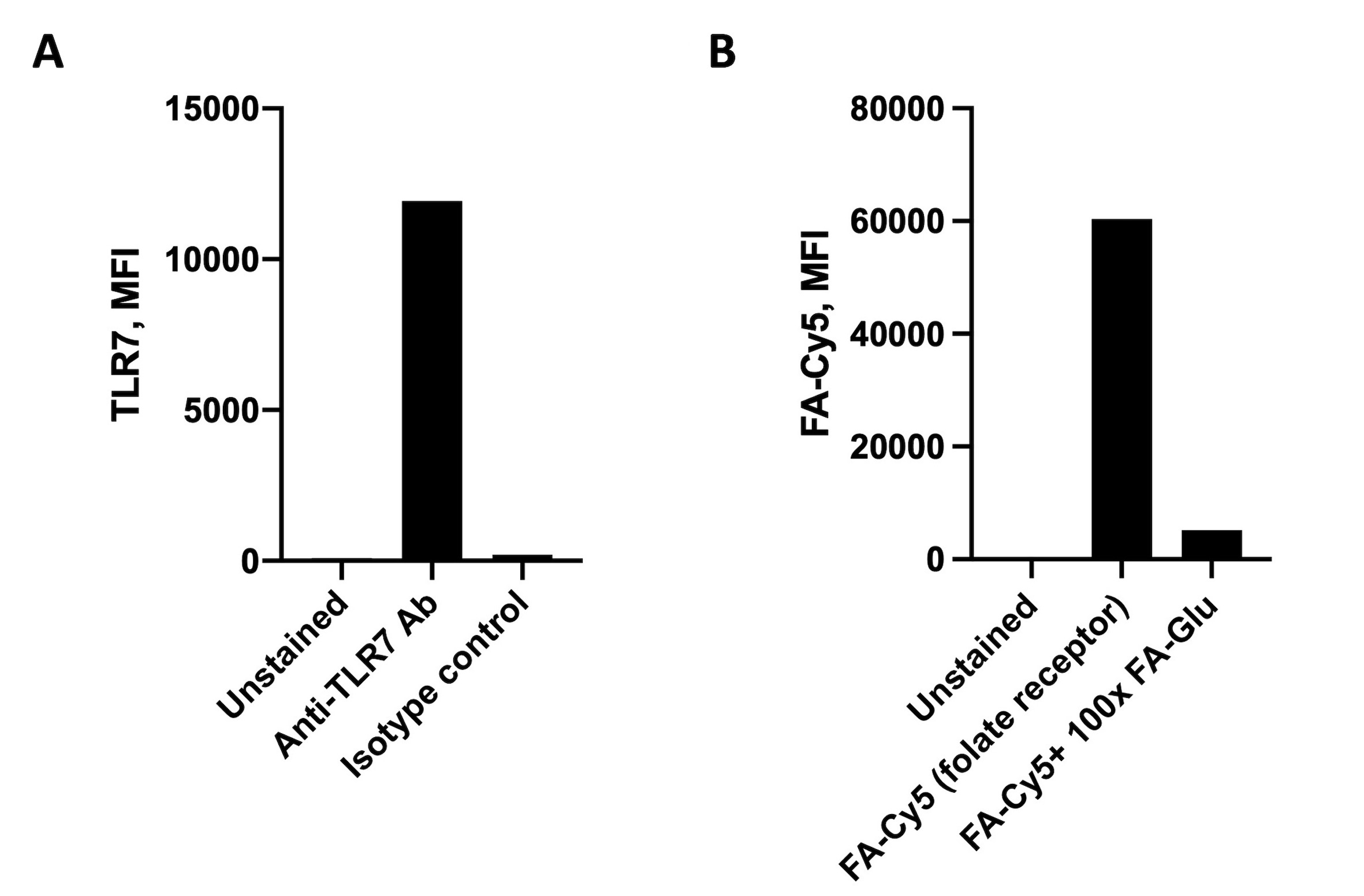

Supplement: Supplementary file 5 [file Image_5.jpeg]

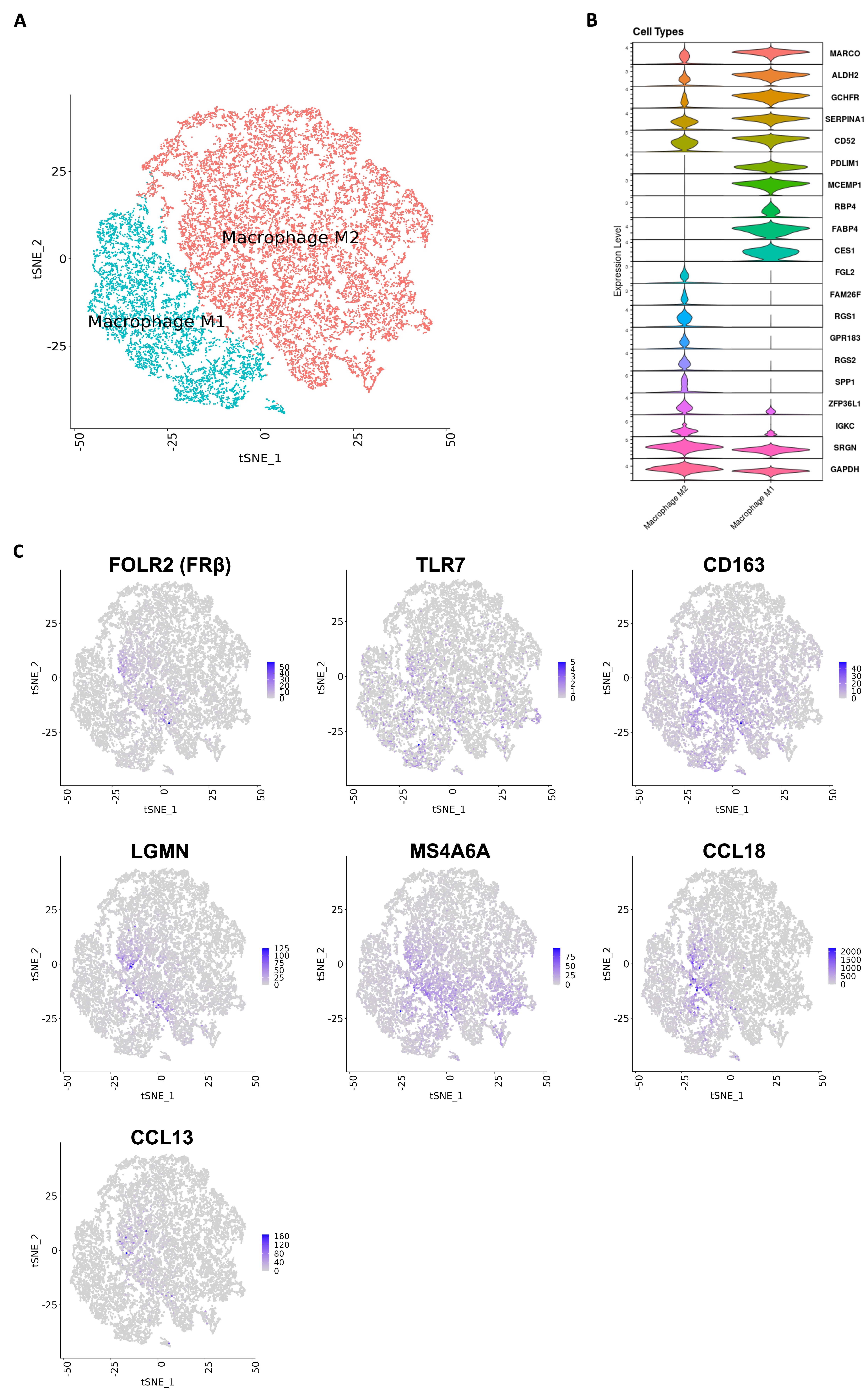

Supplement: Supplementary file 6 [file Image_6.jpeg]

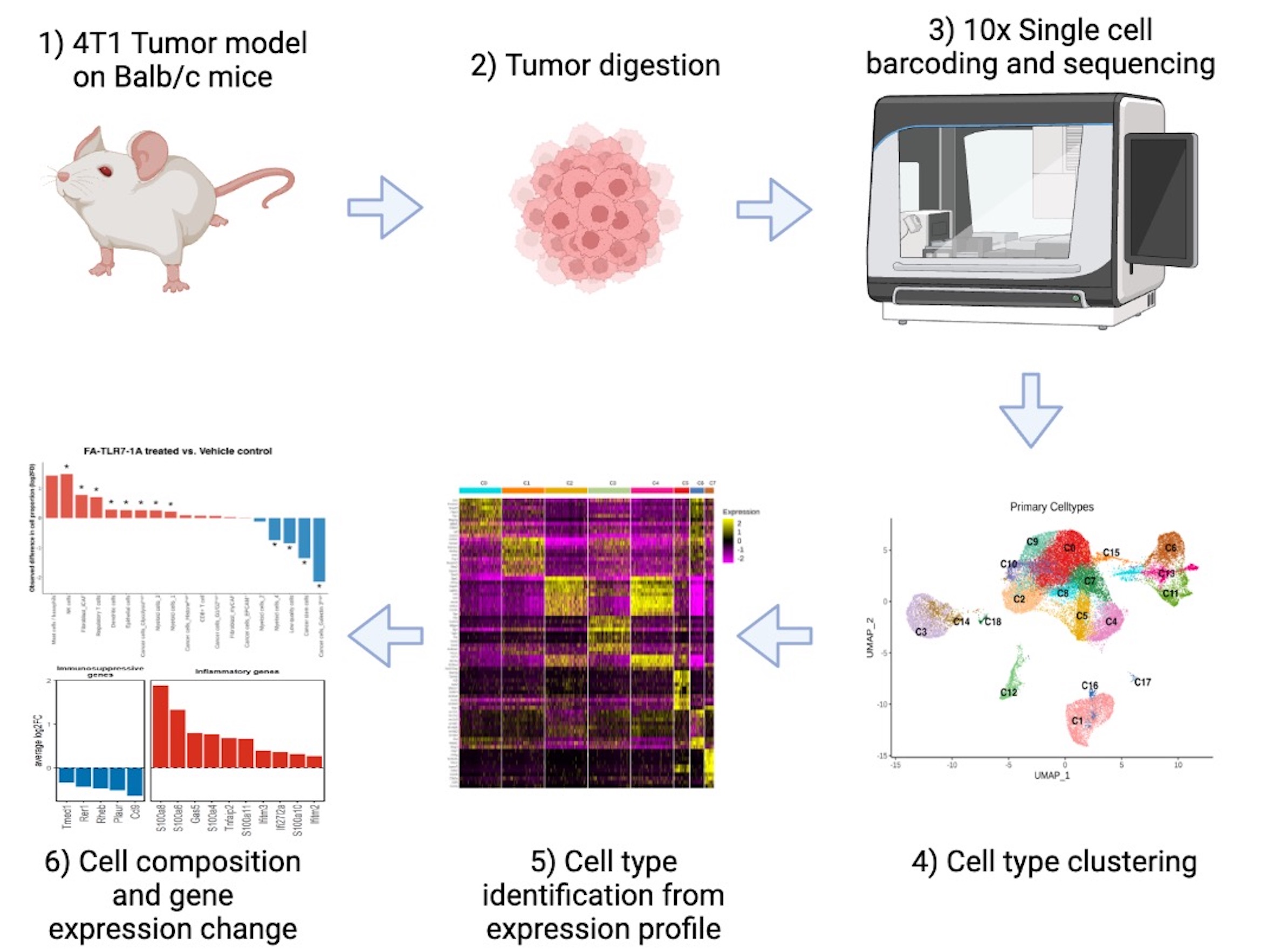

Supplement: Supplementary file 7 [file Image_7.jpeg]

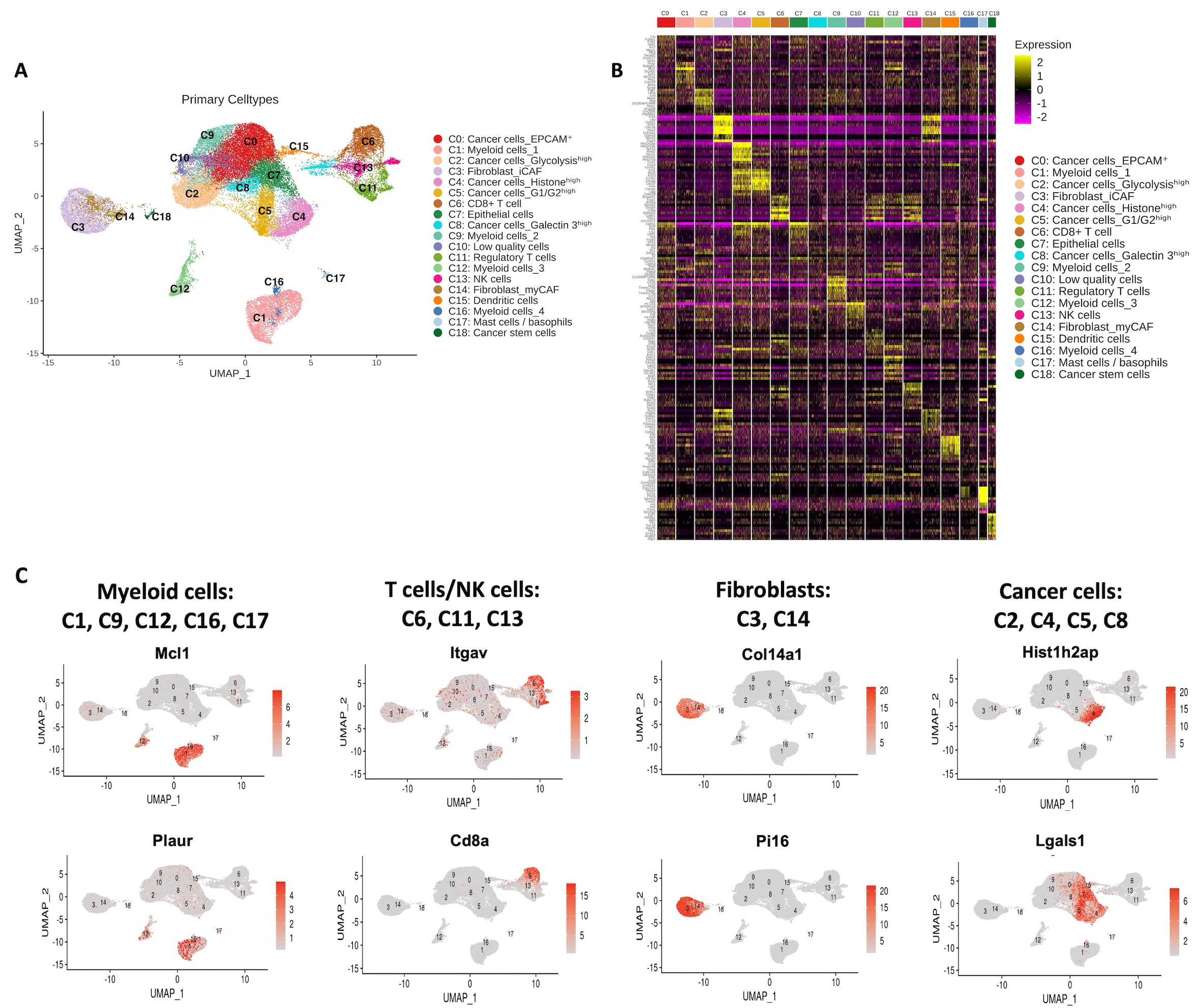

Supplement: Supplementary file 8 [file Image_8.jpeg]

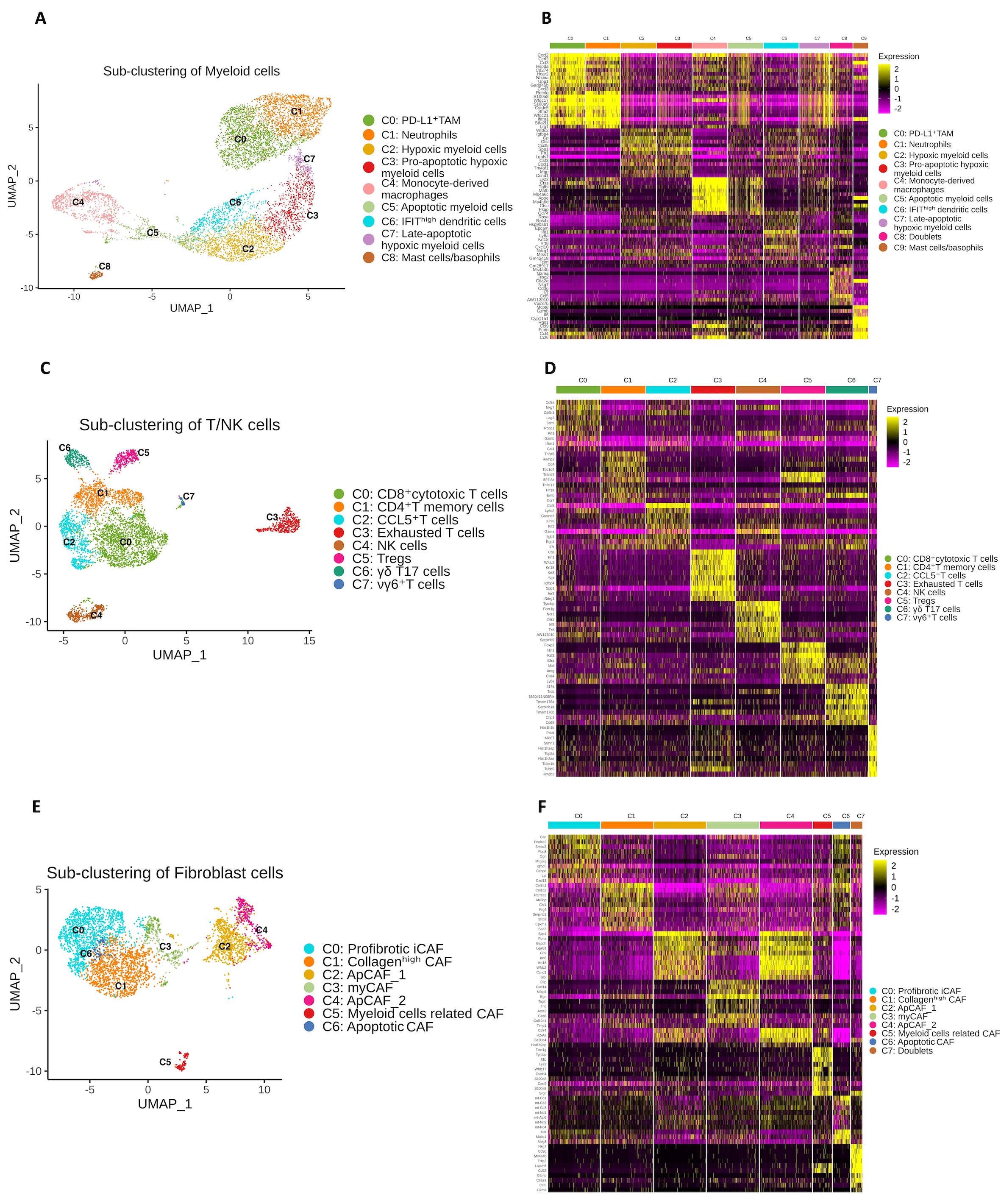

Supplement: Supplementary file 9 [file Image_9.jpeg]
