# Supplementary material for: Tumor-specific activation of folate receptor beta enables reprogramming of immune cells in the tumor microenvironment
Source: Front Immunol. 2024 Feb 7;15:1354735. doi: 10.3389/fimmu.2024.1354735 (PMC10879311; doi:10.3389/fimmu.2024.1354735)
Supplement: Supplementary file 10 [file DataSheet_1.docx]

**Supplemental Figure Legends**

**Fig S1. Analysis of the correlation between FRβ expression and cancer patient survival.** (**A**) Lung squamous cell carcinoma. (**B**) Ovarian serous cystadenocarcinoma. (**C**) Breast invasive carcinoma. FRβ expression and patient survival data were extracted from public TCGA database and analyzed using UALCAN as described in methods. According to UALCAN, high expressions were characterized with TPM values in the upper quartile, and low/medium expression were characterized with TPM values below the upper quartile.

**Fig S2. Gating strategy for analysis of FRβ expression on monocytes in PBMCs and spleens of healthy mice.** PBMCs were prepared from peripheral blood and splenocytes were collected from spleens of healthy mice prior to analysis for FRβ expression, as described in the Method section. Cells were stained with live/dead cell marker Zombie violet and CD45-BV510 and then as follows: 1) CD11b-AF700 and Ly6C-PE for monocytes, 2) FRβ-APC for FRβ protein, and 3) Folate-Cy5 for functional FRβ.

**Fig S3. Gating strategy for analysis of FRβ expression in healthy human PBMCs.** PBMCs were prepared from heathy human peripheral blood (n=5) and analyzed for FRβ expression, as described in the Methods section. Total FRβ and functional FRβ gating strategies for (**A**) monocytes/ macrophages, (**B**) monocyte subtypes, (**C**) dendritic cells, (**D**) neutrophils, (**E**) T cells, (**F**) B cells, (**G**) NK cells, (**H**) basophils are shown here.

**Fig S4. Evaluation of FRβ and TLR7 expression in murine 4T1 tumors.** 4T1 tumors were digested as described in Fig 1A and Methods section. Cells were then stained with live/dead cell marker Zombie violet and CD45-BV510 prior to staining as follows: FRβ-APC for FRβ; CD11b-AF700 and F4/80-AF488 for macrophages; CD3-AF488 for T cells; CD11c-FITC for dendritic cells; EpCAM-FITC for cancer cells; and Thy1-FITC for fibroblasts. Cells were then fixed and permeabilized and further stained with TLR7-PE to assess TLR7 expression. (**A**) FMO gating strategy for macrophage analysis. TLR7 and FRβ expression analysis and FMO gating strategy for (**B**) T cells, (**C**) dendritic cells, (**D**) cancer cells, and (**E**) fibroblasts.

**Fig S5. Analysis of the functional folate receptor and TLR7 expression of Raw264.7 cells.** An FR-positive subclone of the murine macrophage-derived RAW264.7 cell line was obtained as previously described(1) and cultured in folate free RPMI1640 medium. Cells were stained with anti-mouse TLR7 antibody and the relevant isotype control to determine the TLR7 expression (**A**), or stained with FA-Cy5 in the absence or presence of100X FA-Glu to determine the functional folate receptor expression (**B**). Mean fluorescence intensity (MFI) was analyzed by flow cytometry.

**Fig S6.** **Co-localization of FOLR2 (FRβ) with other immunosuppressive genes in macrophages from human lung cancer scRNA-seq data.**Macrophages from published scRNA-seq data(2) were isolated and clustered into M1 and M2 TAM sub-populations (**A**) with the top marker genes from each cluster listed (**B**). (**C**) Co-localization of M2 markers and immunosuppressive genes with FOLR2 (FRβ) are shown. Data were obtained from 8 human lung cancer patients.

**Fig S7. Experimental design for scRNA-seq analysis.** The experimental workflow was as follows: 1) Balb/c mice were implanted with 4T1 tumors and treated with either vehicle control or FA-TLR7-1A, as described in the Methods section. 2) Two weeks after the first FA-TLR7-1A treatment, mice were sacrificed, and tumors were dissociated. 3) Tumor samples were prepared for single-cell RNA sequencing (scRNA-seq) according to protocols recommended by 10x Genomics. 4) The collected scRNA-seq data library was analyzed using Seurat, as described in the Methods section. 5) Cell types were identified through clustering based on differentially expressed marker genes. 6) The composition of cells and gene expression data were analyzed by comparing the FA-TLR7-1A treated group with the vehicle control group. Vehicle control = 3% DMSO in PBS. Figure S5 was created with Biorender.com. Statistically significant changes in population sizes are denoted with *.

**Fig S8**. **Cell clustering and characterization.** (**A**) UMAPs representation of main clustering of all 4T1 tumor cells. (**B**) Heatmap showing the top 10 marker genes for each cluster. (**C**) UMAPs showing marker gene expression for each major cell group.

**Fig S9**. **Sub-clustering and characterization of myeloid cells (A&B), T/NK cells (C&D), and fibroblasts (E&F).** UMAPs representations of the clustering of major cell types from 4T1 tumors are shown in the left panels and heatmaps showing the top 10 marker genes for each major cell type are shown on the right.

**Supplemental Tables**

**Table S1: Staining markers for flow cytometry**

| Corresponding figure | Sample source | Cell type / markers | Antibody |
| --- | --- | --- | --- |
| Figure 1A & 2A-C, S4 | Mouse 4T1 tumor digestion | Macrophages:  Zombie violet-CD45+CD11b+F4/80+,  then stained for FRβ and TLR7 | anti-mouse CD45-BV605,  anti-mouse CD11b-AF700,  anti-mouse F4/80-AF488,  anti-mouse FRβ-APC,  anti-mouse TLR7-PE,  10nM folate-Cy5,  1μM folate-glucosamine as competition |
|  |  | T cells:  Zombie violet-CD45+CD3+,  then stained for FRβ and TLR7 | anti-mouse CD45-BV605,  anti-mouse CD3-AF488,  anti-mouse FRβ-APC,  anti-mouse TLR7-PE |
|  |  | Dendritic cells:  Zombie violet-CD45+CD11c+,  then stained for FRβ and TLR7 | anti-mouse CD45-BV605,  anti-mouse CD11c-FITC,  anti-mouse FRβ-APC,  anti-mouse TLR7-PE |
|  |  | Cancer cells:  Zombie violet-CD45-EpCAM+,  then stained for FRβ and TLR7 | anti-mouse CD45-BV605,  anti-mouse EpCAM-FITC,  anti-mouse FRβ-APC,  anti-mouse TLR7-PE |
|  |  | Fibroblasts:  Zombie violet-CD45-Thy1+,  then stained for FRβ and TLR7 | anti-mouse CD45-BV605,  anti-mouse Thy1-FITC,  anti-mouse FRβ-APC,  anti-mouse TLR7-PE |
| Figure 1B | Mouse monocytes from PBMCs and spleens | Monocytes:  Zombie violet-CD45+CD11b+Ly6C+,  then stained for FRβ | anti-mouse CD45-BV605,  anti-mouse CD11b-PerCP,  anti-mouse Ly6C-PE,  anti-mouse FRβ-APC,  100nM folate-Cy5,  10μM folate-glucosamine as competition |
| Figure 1C, S3 | Healthy human PBMCs  (All cell types were stained with:  anti-human FRβ-PE,  100nM folate-Cy5, and 10μM folate-glucosamine as competition) | T cells:  Zombie violet-CD45+CD3+,  then stained for FRβ | anti-human CD45-BV605, anti-human CD3-PE/Cy7 |
|  |  | B cells:  Zombie violet-CD45+CD3-CD19+, then stained for FRβ | anti-human CD45-BV605,  anti-human CD3-PE/Cy7, anti-human CD19-FITC |
|  |  | NK cells:  Zombie violet-CD45+CD3-CD56+, then stained for FRβ | anti-human CD45-BV605, anti-human CD3-PE/Cy7, anti-human CD56-FITC |
|  |  | Monocyte subtypes:  Zombie violet-CD45+, stained for CD14 & CD16 for monocyte subtypes, and then stained for FRβ | anti-human CD45-BV605, anti-human CD14-FITC  anti-human CD16-PE/Cy5 |
|  |  | Dendritic cells:  Zombie violet-CD45+CD11c+, then stained for FRβ | anti-human CD45-BV605, anti-human CD11c-PE/Cy7 |
|  |  | Monocytes/macrophages:  Zombie violet-CD45+CD11b+CD68+, then stained for FRβ | anti-human CD45-BV605, anti-human CD11b-FITC, anti-human CD68-PE/Cy7 |
|  |  | Basophils:  Zombie violet-CD123+CCR3+, then stained for FRβ | anti-human CD123-FITC, anti-human CCR3-BV605 |
|  |  | Neutrophils (isolated by density gradient centrifugation):  Zombie violet-CD45+, then stained for FRβ | anti-human CD45-BV605 |
| Figure 1D | Human monocyte-derived M2 macrophages | M2-like macrophages:  Zombie violet-CD68+CD206+  Functional FRβ: folate-fluorescein | anti-human CD206-APC,  anti-human CD68-PE,  100nM folate-fluorescein,  10μM folate-glucosamine as competition |

**Table S2: Antibody list for flow cytometry**

| Material name | Catalog number | Vendor |
| --- | --- | --- |
| anti-mouse CD45-BV605 | #103140 | BioLegend |
| anti-mouse CD11b-AF700 | #101222 |  |
| anti-mouse F4/80-AF488 | #123120 |  |
| anti-mouse CD3-AF488 | #100210 |  |
| anti-mouse CD11c-FITC | #117306 |  |
| anti-mouse EpCAM-FITC | #118208 |  |
| anti-mouse Thy1-FITC | #105306 |  |
| anti-mouse FRβ-APC | #153306 |  |
| anti-mouse TLR7-PE | #160004 |  |
| anti-mouse CD11b-PerCP | #101229 |  |
| anti-mouse Ly6C-PE | #128007 |  |
| anti-human CD206-APC | #321109 |  |
| anti-human CD68-PE | #333807 |  |
| anti-human FRβ-PE | #391704 |  |
| anti-human CD45-BV605 | #304042 |  |
| anti-human CD3-PE/Cy7 | #300419 |  |
| anti-human CD19-FITC | #302205 |  |
| anti-human CD56-FITC | #318303 |  |
| anti-human CD14-FITC | #301803 |  |
| anti-human CD16-PE/Cy5 | #302009 |  |
| anti-human CD11c-PE/Cy7 | #301607 |  |
| anti-human CD11b-FITC | #301329 |  |
| anti-human CD68-PE/Cy7 | #333815 |  |
| anti-human CD123-FITC | #306013 |  |
| anti-human CCR3-BV605 | #310715 |  |

**Table S3: Characterization of single cell RNAseq clusters**

| Cell cluster | Subtype definition | Marker genes | References |
| --- | --- | --- | --- |
| Main cluster | C0: Cancer cells_EpCAM^+^ | Epcam, Clu, Fgfbp1, Hes1, Mgst1 | (3-5) |
|  | C1: Myeloid cells_1 | Cd14, Plaur, Ly6g, Nfkbia, Csf3r | (6, 7) |
|  | C2: Cancer cells_Glycolysis^high^ | Pgk1, Ldha, Esd, Aldoa, Bsg | (8, 9) |
|  | C3: Fibroblast_iCAF | Pi16, Lbp, Clec3b, Plpp3, Gpx3 | (10-12) |
|  | C4: Cancer cells_Histone^high^ | Hist1h2ap, Hist1h2ae, Stmn1, Hist1h3c, Hist1h1b | (13, 14) |
|  | C5: Cancer cells_G1/G2^high^ | Epcam, Ccnb2, Cenpa, Cdc20, Cenpe | (15) |
|  | C6: CD8^+^ T cell | Cd3d, Cd3e, CD8a, Cd8b1, Nkg7 |  |
|  | C7: Epithelial cells | Krt8, Epcam, Krt14, Mmp9, Lgals1 | (13) |
|  | C8: Cancer cells_Galectin 3^high^ | Lgals3, Bc1, Snhg1, Il2, Hsp90ab1, Cystm1 | (16, 17) |
|  | C9: Myeloid cells_2 | Spp1, Cxcl5, Lcn2, Tmem176b, Enpp2 | (18) |
|  | C10: Low quality cells | No specific marker genes with high mitochondrial related genes |  |
|  | C11: Regulatory T cells | Foxp3, Il17a, Cd4, Izumo1r, Cd69 | (19) |
|  | C12: Myeloid cells_3 | CD68, Csf1r, CD86, Ly6c2, CD14 |  |
|  | C13: NK cells | Ncr1, Klrb1c, Ifngr1, Klre1, Klra3 | (20) |
|  | C14: Fibroblast_myCAF | Cavin1, Klf6, Col8a1, Tagln, Tnc, Acta2 | (12) |
|  | C15: Dendritic cells | Ifi44, Ifit1, Ifit2, Ifit3, Ifit3b | (21, 22) |
|  | C16: Myeloid cells_4 | S100a9, S100a8, Wfdc17, Cxcl2, IL13 | (23, 24) |
|  | C17: Mast cells / basophils | Mcpt4, Cma1, Slc7a8, Egr3, Hs6st2 | (25, 26) |
|  | C18: Cancer stem cells | Cd34, Egfl7, Ushbp1, Pecam1, Mmrn2 | (27-29) |
| Myeloid cell subclusters | C0: PD-L1^+^ TAM | Cd274, Ccl3, Cxcl2, Ccrl2, Hilpda | (30, 31) |
|  | C1: Neutrophils | Retnlg, S100a8, S100a9, Wfdc21, Cd52 | (32) |
|  | C2: Hypoxic myeloid cells | SPP1, S100a16, Cxcl5,Ctsl, Cxcl1 | (18) |
|  | C3: Pro-apoptotic hypoxic myeloid cells | Similar genes as C2 but contain higher mitochondrial genes | (33) |
|  | C4: Monocyte-derive macrophages | F13a1, Mrc1, Chil3, Mafb, Ms4a6c | (25, 34) |
|  | C5: Apoptotic myeloid cells | Similar genes as C2 but contain the highest mitochondrial genes | (33) |
|  | C6: IFIT^high^ dendritic cells | H2-T22, H2-Eb1, Ifit3, Ifi44, Cxcl10 | (21, 22) |
|  | C7: Late-apoptotic hypoxic myeloid cells | Similar genes as C2 but contain higher mitochondrial genes than C2 and C3 | (33) |
|  | C8: Doublets | Expressed bothe cytotoxic genes (CD8, Nkg7, Gama) and myeloid genes (Ms4a4b, CD247) |  |
|  | C9: Mast cells/basophils | Mcpt8, Gzmb, Cpa3, Il6, Cyp11a1 | (25, 26) |
| T/NK cell subclusters | C0: CD8^+^ cytotoxic T cells | CD8a, CD8b1, Nkg7, Ifitm1, Gzmb | (35) |
|  | C1: CD4^+^ T memory cells | Tnfsf8, Ramp3, CD4 | (19) |
|  | C2: CCL5^+^ T cells | CCl5, Ly6c2, Gramd3, Klhl6, Klf2 |  |
|  | C3: Exhausted T cells | CD8b1, Nkg7, Ccl5, Foxp3, Il2ra | (36, 37) |
|  | C4: NK cells | Tyrobp, Fcer1g, Ncr1, Gzma, Irf8 | (20) |
|  | C5: Tregs | Foxp3, Tnfsfr4, Areg, Ctla4 | (19) |
|  | C6: γδ T17 cells | Il17a, Trdc, Scart2, Tmem176a, Tmem176b | (38, 39) |
|  | C7: Vγ6^+^ T cells | Hist1h1b, Hist1h2ap, Top2a, Mki67, Stmn1 | (39) |
| Fibroblast subclusters | C0: Profibrotic iCAF | Gsn, Pcolce2, Smpd3, Plpp3, Ogn, Mrgprg, Igfbp5, Cebpd, Lpl, Cxcl13 | (10, 12, 40) |
|  | C1: Collagen^high^ CAF | Col3a1, Col1a2, Rarres2, Chl1, Prg4, Serpinb2, Saa3 |  |
|  | C2: ApCAF_1 | Spp1, Lgals1, Krt8, Krt18, Wfdc2, Slpi, Cd74, H2-Aa | (10, 12) |
|  | C3: myCAF | Cilp, Cxcl14, Mfap4, Bgn, Tagln, Tnc, Acta2, Gas6, Col12a1, Timp1 | (10, 12, 41) |
|  | C4: ApCAF_2 | Same as cluster 2, but all marker genes are upregulated comparing to cluster 2 |  |
|  | C5: Myeloid cells related CAF | Fcer1g, Il1b, Lyz2, Wfdc17, Cstdc4, S100a8, Cxcl2, S100a9, Srgn | (10, 42) |
|  | C6: Apoptotic CAF | mt-Co1, mt-Co2, mt-Co3, mt-Nd1, mt-Atp6, mt-Nd2, Md-Nd4, Xist, Malat1, Meg3 | (33) |
|  | C7: Doublets | Nkg7, Cd3g, Ms4a4b, Trbc2, Laptm5, Cd52, Gzmb, Ctla2a, Ccl5 |  |

**References:**

1. Lu Y, Stinnette TW, Westrick E, Klein PJ, Gehrke MA, Cross VA, et al. Treatment of experimental adjuvant arthritis with a novel folate receptor-targeted folic acid-aminopterin conjugate. Arthritis Res Ther. 2011;13(2):R56.

2. Qian J, Olbrecht S, Boeckx B, Vos H, Laoui D, Etlioglu E, et al. A pan-cancer blueprint of the heterogeneous tumor microenvironment revealed by single-cell profiling. Cell Res. 2020;30(9):745-62.

3. Gilbert S, Dominic F, Martin W, Christian E, Peter O, Carina H, et al. EpCAM expression in primary tumour tissues and metastases: an immunohistochemical analysis. Journal of Clinical Pathology. 2011;64(5):415.

4. Shannan B, Seifert M, Leskov K, Willis J, Boothman D, Tilgen W, et al. Challenge and promise: roles for clusterin in pathogenesis, progression and therapy of cancer. Cell Death Differ. 2006;13(1):12-9.

5. Zhang Z, Liu M, Hu Q, Xu W, Liu W, Sun Q, et al. FGFBP1, a downstream target of the FBW7/c-Myc axis, promotes cell proliferation and migration in pancreatic cancer. Am J Cancer Res. 2019;9(12):2650-64.

6. Zhang L, Li Z, Skrzypczynska KM, Fang Q, Zhang W, O'Brien SA, et al. Single-Cell Analyses Inform Mechanisms of Myeloid-Targeted Therapies in Colon Cancer. Cell. 2020;181(2):442-59.e29.

7. Veglia F, Hashimoto A, Dweep H, Sanseviero E, De Leo A, Tcyganov E, et al. Analysis of classical neutrophils and polymorphonuclear myeloid-derived suppressor cells in cancer patients and tumor-bearing mice. J Exp Med. 2021;218(4).

8. Gojo J, Englinger B, Jiang L, Hübner JM, Shaw ML, Hack OA, et al. Single-Cell RNA-Seq Reveals Cellular Hierarchies and Impaired Developmental Trajectories in Pediatric Ependymoma. Cancer Cell. 2020;38(1):44-59.e9.

9. Darmanis S, Sloan SA, Croote D, Mignardi M, Chernikova S, Samghababi P, et al. Single-Cell RNA-Seq Analysis of Infiltrating Neoplastic Cells at the Migrating Front of Human Glioblastoma. Cell Rep. 2017;21(5):1399-410.

10. Elyada E, Bolisetty M, Laise P, Flynn WF, Courtois ET, Burkhart RA, et al. Cross-Species Single-Cell Analysis of Pancreatic Ductal Adenocarcinoma Reveals Antigen-Presenting Cancer-Associated Fibroblasts. Cancer Discov. 2019;9(8):1102-23.

11. Han C, Liu T, Yin R. Biomarkers for cancer-associated fibroblasts. Biomark Res. 2020;8(1):64.

12. Kieffer Y, Hocine HR, Gentric G, Pelon F, Bernard C, Bourachot B, et al. Single-Cell Analysis Reveals Fibroblast Clusters Linked to Immunotherapy Resistance in Cancer. Cancer Discov. 2020;10(9):1330-51.

13. Lee RD, Munro SA, Knutson TP, LaRue RS, Heltemes-Harris LM, Farrar MA. Single-cell analysis identifies dynamic gene expression networks that govern B cell development and transformation. Nat Commun. 2021;12(1):6843.

14. Song S, Perez JV, Svitko W, Ricketts MD, Dean E, Schultz D, et al. Rap1-mediated nucleosome displacement can regulate gene expression in senescent cells without impacting the pace of senescence. Aging Cell. 2020;19(1):e13061.

15. Wu T, Zhang X, Huang X, Yang Y, Hua X. Regulation of cyclin B2 expression and cell cycle G2/m transition by menin. J Biol Chem. 2010;285(24):18291-300.

16. Wang H, Deng G, Ai M, Xu Z, Mou T, Yu J, et al. Hsp90ab1 stabilizes LRP5 to promote epithelial-mesenchymal transition via activating of AKT and Wnt/β-catenin signaling pathways in gastric cancer progression. Oncogene. 2019;38(9):1489-507.

17. Caputo S, Grioni M, Brambillasca CS, Monno A, Brevi A, Freschi M, et al. Galectin-3 in Prostate Cancer Stem-Like Cells Is Immunosuppressive and Drives Early Metastasis. Front Immunol. 2020;11:1820.

18. Wei J, Chen Z, Hu M, He Z, Jiang D, Long J, et al. Characterizing Intercellular Communication of Pan-Cancer Reveals SPP1+ Tumor-Associated Macrophage Expanded in Hypoxia and Promoting Cancer Malignancy Through Single-Cell RNA-Seq Data. Front Cell Dev Biol. 2021;9:749210.

19. Miragaia RJ, Gomes T, Chomka A, Jardine L, Riedel A, Hegazy AN, et al. Single-Cell Transcriptomics of Regulatory T Cells Reveals Trajectories of Tissue Adaptation. Immunity. 2019;50(2):493-504.e7.

20. Crinier A, Milpied P, Escalière B, Piperoglou C, Galluso J, Balsamo A, et al. High-Dimensional Single-Cell Analysis Identifies Organ-Specific Signatures and Conserved NK Cell Subsets in Humans and Mice. Immunity. 2018;49(5):971-86.e5.

21. Jang J-S, Lee J-H, Jung N-C, Choi S-Y, Park S-Y, Yoo J-Y, et al. Rsad2 is necessary for mouse dendritic cell maturation via the IRF7-mediated signaling pathway. Cell Death & Disease. 2018;9(8):823.

22. Zhang J, Sze DM, Yung BY, Tang P, Chen WJ, Chan KH, et al. Distinct expression of interferon-induced protein with tetratricopeptide repeats (IFIT) 1/2/3 and other antiviral genes between subsets of dendritic cells induced by dengue virus 2 infection. Immunology. 2016;148(4):363-76.

23. Zhao F, Hoechst B, Duffy A, Gamrekelashvili J, Fioravanti S, Manns MP, et al. S100A9 a new marker for monocytic human myeloid-derived suppressor cells. Immunology. 2012;136(2):176-83.

24. Alshetaiwi H, Pervolarakis N, McIntyre LL, Ma D, Nguyen Q, Rath JA, et al. Defining the emergence of myeloid-derived suppressor cells in breast cancer using single-cell transcriptomics. Sci Immunol. 2020;5(44).

25. Cohen M, Giladi A, Gorki AD, Solodkin DG, Zada M, Hladik A, et al. Lung Single-Cell Signaling Interaction Map Reveals Basophil Role in Macrophage Imprinting. Cell. 2018;175(4):1031-44.e18.

26. Shi S, Ye S, Mao J, Ru Y, Lu Y, Wu X, et al. CMA1 is potent prognostic marker and associates with immune infiltration in gastric cancer. Autoimmunity. 2020;53(4):210-7.

27. Sidney LE, Branch MJ, Dunphy SE, Dua HS, Hopkinson A. Concise review: evidence for CD34 as a common marker for diverse progenitors. Stem Cells. 2014;32(6):1380-9.

28. Baumann CI, Bailey AS, Li W, Ferkowicz MJ, Yoder MC, Fleming WH. PECAM-1 is expressed on hematopoietic stem cells throughout ontogeny and identifies a population of erythroid progenitors. Blood. 2004;104(4):1010-6.

29. Nichol D, Stuhlmann H. EGFL7: a unique angiogenic signaling factor in vascular development and disease. Blood. 2012;119(6):1345-52.

30. Ho DW, Tsui YM, Chan LK, Sze KM, Zhang X, Cheu JW, et al. Single-cell RNA sequencing shows the immunosuppressive landscape and tumor heterogeneity of HBV-associated hepatocellular carcinoma. Nat Commun. 2021;12(1):3684.

31. Liu C, Zhou X, Zeng H, Wu D, Liu L. HILPDA Is a Prognostic Biomarker and Correlates With Macrophage Infiltration in Pan-Cancer. Front Oncol. 2021;11:597860.

32. Lummertz da Rocha E, Rowe RG, Lundin V, Malleshaiah M, Jha DK, Rambo CR, et al. Reconstruction of complex single-cell trajectories using CellRouter. Nat Commun. 2018;9(1):892.

33. Márquez-Jurado S, Díaz-Colunga J, das Neves RP, Martinez-Lorente A, Almazán F, Guantes R, et al. Mitochondrial levels determine variability in cell death by modulating apoptotic gene expression. Nat Commun. 2018;9(1):389.

34. Yadav MK, Inoue Y, Nakane-Otani A, Tsunakawa Y, Jeon H, Samir O, et al. Transcription factor MafB is a marker of tumor-associated macrophages in both mouse and humans. Biochem Biophys Res Commun. 2020;521(3):590-5.

35. Kiniry BE, Hunt PW, Hecht FM, Somsouk M, Deeks SG, Shacklett BL. Differential Expression of CD8(+) T Cell Cytotoxic Effector Molecules in Blood and Gastrointestinal Mucosa in HIV-1 Infection. J Immunol. 2018;200(5):1876-88.

36. Conde E, Casares N, Mancheño U, Elizalde E, Vercher E, Capozzi R, et al. FOXP3 expression diversifies the metabolic capacity and enhances the efficacy of CD8 T cells in adoptive immunotherapy of melanoma. Mol Ther. 2023;31(1):48-65.

37. Lutz V, Hellmund VM, Picard FSR, Raifer H, Ruckenbrod T, Klein M, et al. IL18 Receptor Signaling Regulates Tumor-Reactive CD8+ T-cell Exhaustion via Activation of the IL2/STAT5/mTOR Pathway in a Pancreatic Cancer Model. Cancer Immunol Res. 2023;11(4):421-34.

38. Spidale NA, Sylvia K, Narayan K, Miu B, Frascoli M, Melichar HJ, et al. Interleukin-17-Producing γδ T Cells Originate from SOX13(+) Progenitors that Are Independent of γδTCR Signaling. Immunity. 2018;49(5):857-72.e5.

39. Tan L, Sandrock I, Odak I, Aizenbud Y, Wilharm A, Barros-Martins J, et al. Single-Cell Transcriptomics Identifies the Adaptation of Scart1(+) Vγ6(+) T Cells to Skin Residency as Activated Effector Cells. Cell Rep. 2019;27(12):3657-71.e4.

40. Thomas D, Radhakrishnan P. Role of Tumor and Stroma-Derived IGF/IGFBPs in Pancreatic Cancer. Cancers (Basel). 2020;12(5).

41. Yu B, Chen X, Li J, Qu Y, Su L, Peng Y, et al. Stromal fibroblasts in the microenvironment of gastric carcinomas promote tumor metastasis via upregulating TAGLN expression. BMC Cell Biol. 2013;14:17.

42. Lin W, Noel P, Borazanci EH, Lee J, Amini A, Han IW, et al. Single-cell transcriptome analysis of tumor and stromal compartments of pancreatic ductal adenocarcinoma primary tumors and metastatic lesions. Genome Medicine. 2020;12(1):80.
